# β_2_-integrin LFA1 mediates airway damage following neutrophil transepithelial migration during respiratory syncytial virus infection

**DOI:** 10.1183/13993003.02216-2019

**Published:** 2020-08-06

**Authors:** Jenny Amanda Herbert, Yu Deng, Pia Hardelid, Elisabeth Robinson, Luo Ren, Dale Moulding, Rosalind Louise Smyth, Claire Mary Smith

**Affiliations:** 1 UCL Great Ormond Street Institute of Child Health, London, UK; 2 Dept of Respiratory Medical Centre, Chongqing Key Laboratory of Child Infection and Immunity, Children's Hospital of Chongqing Medical University, China International Science and Technology Cooperation Base of Child Development and Critical Disorders, Ministry of Education Key Laboratory of Child Development and Disorders, Chongqing, China; 3 Joint senior author

## Abstract

Respiratory syncytial virus (RSV) bronchiolitis is the most common cause of infant hospital admissions, but there is limited understanding of the mechanisms of disease, and no specific antiviral treatment. Using a novel *in vitro* primary transepithelial neutrophil migration model and innovative imaging methods, we show that RSV infection of nasal airway epithelium increased neutrophil transepithelial migration and adhesion to infected epithelial cells, which is associated with epithelial cell damage and reduced ciliary beat frequency, but also with a reduction in infectious viral load.

Following migration, RSV infection results in greater neutrophil activation, degranulation and release of neutrophil elastase into the airway surface media compared to neutrophils that migrated across mock-infected nasal epithelial cells. Blocking of the interaction between the ligand on neutrophils (the β_2_-integrin LFA-1) for intracellular adhesion molecule (ICAM)-1 on epithelial cells reduced neutrophil adherence to RSV-infected cells and epithelial cell damage to pre-infection levels, but did not reduce the numbers of neutrophils that migrated or prevent the reduction in infectious viral load.

These findings have provided important insights into the contribution of neutrophils to airway damage and viral clearance, which are relevant to the pathophysiology of RSV bronchiolitis. This model is a convenient, quantitative preclinical model that will further elucidate mechanisms that drive disease severity and has utility in antiviral drug discovery.

## Abstract 2

**Neutrophils reduce RSV load, but their adherence to airway epithelial cells *via* β_2_-integrin LFA1 inflicts collateral airway damage** http://bit.ly/38ZOIn7

## Introduction

Respiratory syncytial virus (RSV) is the leading cause of bronchiolitis and the most prevalent viral cause of hospitalisation in children aged <1 year [1]. There is currently no vaccine to prevent RSV infection and no specific antiviral treatment. Recent advances in structural biology have revived RSV vaccine and antiviral development, with several vaccines [2] and antiviral candidates [3–5] coming through the therapeutic pipeline. Expanding our understanding of the mechanisms that underlie the pathophysiology of RSV bronchiolitis is important to support the development of RSV-specific therapies. Studies using *in vitro* human ciliated airway epithelial cell models of RSV infection have led to important insights into host responses to respiratory viruses [6–9]. However, unlike lung tissue from infants with RSV [10, 11], these *in vitro* models reveal few signs of cytopathology during RSV infection, which raises doubt about their utility when studying the pathophysiology of RSV bronchiolitis in infants.

**FIGURE 1:**
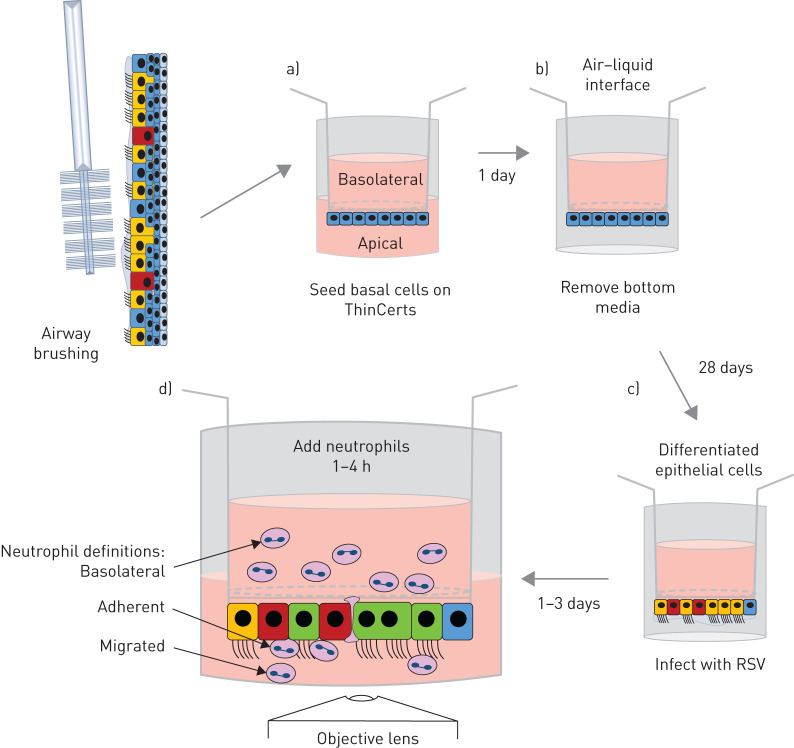
Schematic diagram of primary human nasal airway epithelial cell neutrophil migration model. a) Primary nasal airway epithelial basal cells were seeded onto the underside of a 3 µm pore size polyethylene terephthalate ThinCert membrane inserts and allowed to attach for 4 h. Membrane inserts were subsequently inverted and maintained in media to allow a confluent epithelial monolayer to develop for 1 day. b) Membrane inserts were exposed to an air–liquid interface and allowed to fully differentiate for 28 days. c) Membrane inserts were inverted and infected apically with green fluorescent protein respiratory syncytial virus (RSV) or mock-infected for 2 h and the infection allowed to progress for 24 or 72 h. d) Ultrapure neutrophils isolated from venous blood were added to the basolateral side of the membrane inserts, and were allowed to migrate for 1 or 4 h. Outcome measures are identified.

Neutrophils are the predominant immune cell recruited to the lungs of infants with RSV bronchiolitis [12, 13]. Their role in host defence is not fully understood. We hypothesised that migration of neutrophils across RSV-infected nasal airway epithelial cells (nAECs) contributes to cellular damage, and reveal important host response mechanisms. We previously developed a neutrophil migration model [14, 15] using a human alveolar type II cell line (A549), which is commonly used to study RSV infection *in vitro* [15–17]. However, ciliated airway epithelial cells are the main target for RSV infection and immortalised cell lines often lack appropriate cell polarisation and many other important properties found in the airway, such as mucus. Therefore, in order to interrogate neutrophil transepithelial migration further, we have developed a more physiologically relevant *in vitro* model using primary human nasal epithelial cells grown at the air–liquid interface (ALI) (figure 1). Primary airway epithelial cells are seeded on the underside of porous membrane inserts, rather than the topside as in conventional ALI culture. This is because, although there is some suggestion that neutrophil migration can occur against gravity, our preliminary studies indicated that the numbers of neutrophils recovered is very low (∼2500 cells). Our gravity-fed system has been demonstrated to be an ideal system to study neutrophil function following transepithelial migration. We observed neutrophil chemotaxis across primary differentiated nAECs and, for the first time, we measured neutrophil adherence and the associated epithelial damage, including ciliary beat frequency, a sensitive assessment of cellular toxicity.

## Materials and methods

### Participants

Peripheral blood and airway epithelial cells were obtained from healthy adult donors at University College London (UCL) Great Ormond Street Institute of Child Health (London, UK). Written informed consent was obtained from all donors prior to their enrolment in the study. Study approval was obtained from the UCL research ethics committee (4735/002). All methods were performed in accordance with the relevant guidelines and regulations.

### Virus purification and quantification

A recombinant RSV A2 strain that possesses a green fluorescent protein (GFP)-tagged large (L) polymerase, which expresses GFP in epithelial cells that contain replicating virus, was kindly provided by Fix *et al.* [18]. Viral stock preparation and quantification of viral titre from serially diluted nAEC supernatants was performed using HEp-2 cells (ATCC CCL-23), as described previously [14, 15]. Quantitative reverse-transcriptase PCR was performed using TaqMan Universal Master Mix II, with UNG (Applied Biosystems, California, USA). A total volume of 20 μL was used with 1 μL of cDNA per reaction. Primers, probes and reaction conditions were used as previously described [14, 19]. A plasmid containing the N protein sequence [20] was used to quantify the N protein copy number in nAEC supernatant and from within cells. RSV load was extrapolated from a standard curve of known N protein copies.

### Neutrophil transepithelial migration assay

Primary airway epithelial cells were obtained from nasal brushings, as described previously [21] or purchased from Epithelix Sàrl (Geneva, Switzerland). A diagram of our methodology is shown in figure 1 and a detailed protocol for nAEC culture can be found in the supplementary methods. We found no difference in the cultures grown from a brushing or from commercial supplier (supplementary figure S1). Briefly, progenitor basal cells were propagated in co-culture with 3T3-JF mouse fibroblast feeder layers, as described previously [22]. Primary nAECs are seeded into feeder layers at a density of 5×10^5^ per flask in F-media (DMEM and Hams F12 media at a 3:1 ratio supplemented with 1×penicillin streptomycin, 7.5% fetal bovine serum (Gibco, Waltham, MA, USA), 5 mM Y-27632 (Abcam, Cambridge, UK), 25 ng·mL^−1^ hydrocortisone/0.125 ng·mL^−1^ epidermal growth factor (Sigma, St Louis, MO, USA), 5 mg·mL^−1^ insulin (Sigma), 0.1 nM cholera toxin (Sigma) and amphotericin B (2.50 μg·mL^−1^)) and cultured until confluent. Primary nAECs and 3T3-J2 fibroblasts were then separated by differential trypsin dissociation, as previously described [22]. Basal cells were collected in fresh F-media and centrifuged at 200×*g* for 3 min.

Collagen-coated 3-μm pore membrane inserts (ThinCert; Greiner, Kremsmunster, Austria, with culture surface of 33.6 mm^2^) were inverted and 300 000 cells·cm^−2^ nAECs in 70 μL of F-media were seeded onto the bottom of the insert and incubated at 37°C 5% carbon diolxide (CO_2_) for 4–6 h. After incubation, membrane inserts were inverted into a 24-well plate and 500 μL of fresh F-media added underneath and 100 μl of F-media supplemented with 30 μg·mL^−1^ collagen I and 5% (v/v) Matrigel (Corning, Corning, NY, USA) added to the top of the membrane inserts. Cells were incubated for 24–48 h at 37°C 5% CO_2._ After this period, cells were placed at ALI. Media was aspirated from both sides of the membrane insert and cells fed basolaterally with 100 μL of ALI media (1:1 DMEM: airway epithelial cell growth media (PromoCell, Heidelberg, Germany), with all supplements added, further supplemented with 2.5 μg·mL^−1^ Amphotericin B, 1×penicillin/streptomycin and 1 μm retinoic acid (Sigma). The medium was replaced every 1–2 days, and cells incubated at 37°C 5% CO_2_ in a high-humidity incubator for 4 weeks/28 days to allow cellular differentiation. Once differentiated, cells were infected with RSV as before [19].

Neutrophils were purified from 10 mL of peripheral venous blood using a negative immunoselection neutrophil isolation kit (Stemcell Technologies, Vancouver, Canada), as per the manufacturer's instructions. The mean number of neutrophils isolated from was 1.5×10^7^ cells with a purity of 99.8–99.9% as confirmed by flow cytometry (supplementary figure S1d). Neutrophils were stained with CellTrace Calcein Red-Orange cell stain (ThermoFisher, Waltham, MA, USA), as described previously [14].

For neutrophil transepithelial migration 400 μL of apical surface media was placed underneath the membrane insert for each experimental group: mock infected, RSV infected, mock infected exposed to apical surface media collected from RSV infected cells or N-formylmethionine-leucyl-phenylalanine (fMLP). To investigate the interaction of the integrin leukocyte function-associated antigen-1F (LFA1) with the intracellular cell adhesion molecule ICAM-1, we supplemented the apical surface media collected from RSV-infected cells with 1 μM (2E)-1-(4-acetyl-1-piperazinyl)-3-[4-[[2-(1-methylethyl)phenyl]thio]-3-nitrophenyl]-2-propen-1-one (A286982, TOCRIS BioTechne, Minneapolis, MN, USA), a potent antagonist (inhibitor) of the LFA-1 CD11a I domain [23]. A median inhibitory concentration of 35–44 nM was used, based on that used in other studies [24, 25]. Neutrophils (5×10^5^) were added to the basolateral side of membrane inserts and left to migrate for 1 or 4 h. After migration, neutrophils were collected from the apical side of the epithelial cells for quantification (see later). Apical surface media (containing epithelial secreted factors including cytokines) were collected and membrane inserts were fixed and stained for ICAM-1, acetylated tubulin (cilia) or adherent neutrophils (see later).

### Definitions

Basolateral: the neutrophils that remain on the basolateral side of the epithelium (top chamber) and do not transmigrate across the epithelium. Migrated: the neutrophils that transmigrate across the epithelium and detach into apical surface media (bottom chamber). Adherent neutrophils: the neutrophils that transmigrate across the epithelium and remain adhered to the airway epithelial cells (figure 1).

### ICAM1 expression analysis

ICAM1 expression levels on ciliated nAECs were quantified after RSV infection for 24 and 72 h. Detailed methods on the staining procedure are provided in the supplementary material. Z-stacks were acquired on a confocal microscope (Zeiss LSM710, Oberkochen, Germany) using a ×40 objective with 5 μm distance between each image and up to 50 μm range.

### Quantification of migrated and adherent neutrophils

The number of migrated neutrophils was quantified as described previously [14]. Flow cytometric analysis of CD11b expression on migrated and basolateral neutrophils was performed as described previously [14]. Images of adherent neutrophils were acquired on a confocal microscope (Zeiss LSM710) under a ×40 objective. Neutrophils were counted using the ImageJ counting tool.

### Quantification of epithelial damage

Cell damage was quantified by transepithelial electrical resistance (TEER), red dextran permeability, lactate dehydrogenase (LDH) release and by counting the number of epithelial cells remaining on membrane inserts, as described previously [14], and in supplementary figure S2. To determine ciliary beat frequency (CBF), plates were placed in an incubation chamber (37°C, 5% CO_2_) attached to an inverted microscope system, as described previously [9]. Videos were recorded using a ×20 objective and complementary metal oxide semiconductor digital video camera (Hamamatsu, Shizuoka, Japan) at a rate of 198 frames per second (an example video of a ciliated area is shown in video 1). For each condition, 12 areas per membrane insert were videoed. CBF (Hz) was calculated by fast Fourier transformation using ciliaFA software [26].

### Statistical analysis

Differences between the same donor cells exposed to different test conditions were analysed by paired t-test. A paired two-way ANOVA with Bonferroni correction was used when multiple comparisons were performed (GraphPad Prism v4.0; San Diego, CA, USA). Validation of the tests used and data modelling was performed using StataSE 15 (StataCorp, College Station, TX, USA) (supplementary statistics file).

## Results

Our model (figure 1) generated similar findings at both 24 h and 72 h post-RSV infection and in the figures we show both time points, but in the interest of readability and clarity, in the text, we refer only to the data collected 72 h post-infection. Data on RSV infection of primary ciliated nAECs without neutrophil migration can be found in supplementary figure S2.

### RSV infection increases the number of neutrophils that migrate across ciliated nAECs and dissociate into the apical surface media

**FIGURE 2:**
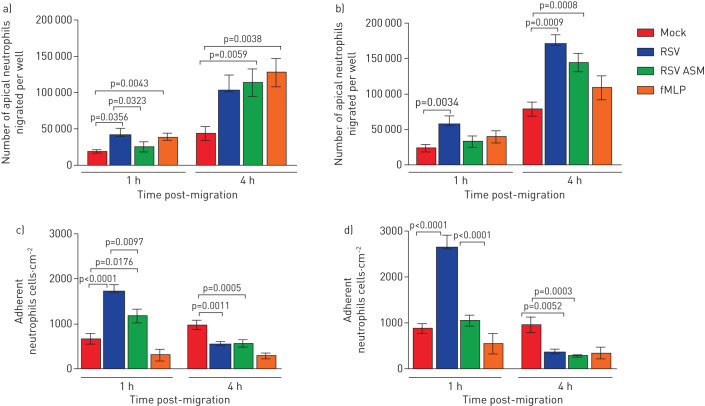
Respiratory syncytial virus (RSV) increases the numbers of neutrophils that migrate across infected nasal epithelial cell cultures. a) The numbers of apical neutrophils that migrated across RSV-infected airway epithelial cell cultures and were released into the apical surface media after 24 h infection. b) The numbers of apical neutrophils that migrated across RSV-infected airway epithelial cell cultures and were released into the apical surface media after 72 h infection. Data are presented as mean±sem of n=6 epithelial donors, n=6 heterologous blood donors. c) The number of neutrophils attached to mock- or RSV-infected human nasal ciliated epithelial cells after 24 h infection. d) The number of neutrophils attached to mock- or RSV-infected human nasal ciliated epithelial cells after 72 h infection. Neutrophil concentrations were quantified in the apical surface media using a plate reader and read against a standard curve. Adhered neutrophils were counted using ImageJ counting tool, the average number of neutrophils from all images is shown (five images per well from n=3 epithelial donors, n=2 heterologous blood donors and n=1 homologous blood donor). Bars represent the mean±sem for cultures mock-infected, RSV-infected, mock-infected and exposed to apical surface media (ASM) collected from RSV-infected cells or mock-infected and exposed apically to the chemoattractant N-formylmethionine-leucyl-phenylalanine (fMLP). Statistical comparison between all groups was performed using a paired t-test. Statistical significance is shown.

Using our differentiated primary airway nasal epithelial cell model (figure 1), we examined the numbers of neutrophils that migrated across and dissociated from the epithelium by measuring the number of fluorescently labelled neutrophils in the apical surface media. We found that after 1 h more than two-fold more neutrophils migrated across ciliated epithelium infected with RSV for 72 h with a mean±sem of neutrophils 5.7×10^4^±1.2×10^4^ migrated per well compared to the mock-infected epithelium (2.3×10^4^±5.2×10^3^) (p=0.034) (figure 2b) (24 h data shown in figure 2a). This is equivalent to 11.4% of the total neutrophils added for RSV-infected epithelial cells and 4.6% for mock-infected epithelial cells. After 4 h of neutrophil migration, we found that 34% of neutrophils had migrated across epithelial cells infected with RSV for 72 h (1.7×10^5^±1.3×10^4^), which was, again, more than two-fold the number of neutrophils that migrated across mock-infected epithelium (15.8%) (7.9×10^4^±9×10^3^) (p=0.0009) (figure 2b).

To investigate whether secreted factors from RSV-infected epithelial cells were contributing to this increased migration of neutrophils, we measured the number of neutrophils that migrated across mock-infected epithelium incubated with apical surface media collected from RSV-infected epithelial cells. We found no difference in the number of migrated neutrophils 1 h after neutrophil migration across mock-infected epithelium compared to mock-infected epithelial cells exposed to apical surface media from cells infected with RSV 72 h post-infection (figure 2b). However, 4 h after migration across mock-infected epithelial cells exposed to apical surface media from cells infected with RSV for 72 h, 1.4×10^5^±1.3×10^4^ cells had migrated, which was 1.7-fold greater than across mock-infected cells exposed to media alone (7.9×10^4^±9×10^3^) (p=0.0008).

### RSV increases the number of neutrophils that remain adherent to the infected ciliated epithelium

We found that after 1 h neutrophil migration the number (mean±sem) of neutrophils adherent to 72 h RSV-infected epithelial cells (2.6×10^3^±2.7×10^2^ neutrophils·cm^−2^) was more than three-fold greater than the mock-infected epithelium (8.6×10^2^ ±1×10^2^ neutrophils·cm^−2^) (p<0.0001) (figure 2d), with more adherent neutrophils at 72 h after RSV infection, compared with that after 24 h of RSV infection (figure 2c) (p<0.05). Interestingly, we found that the number of adherent neutrophils was less 4 h after neutrophil migration (3.6×10^2^±7.4×10^1^ neutrophils·cm^−2^) (figure 2d) compared to 1 h (2.6×10^3^ ±2.7×10^2^ neutrophils·cm^−2^) (p<0.0001), with similar differences 24 h after RSV infection. This was specific to RSV-infected epithelium, as mock-infected epithelium where the number of adherent neutrophils after 4 h neutrophil migration (8.8×10^2^±1×10^2^ neutrophils·cm^−2^) was at similar levels to that found at 1 h (9.2×10^2^±1×10^2^ neutrophils·cm^−2^). To investigate whether secreted factors from RSV-infected epithelial cells were contributing to this increased neutrophil adherence, we measured the number of neutrophils that adhered to mock-infected epithelium incubated with apical surface media collected from RSV-infected epithelial cells. We found that the addition of apical surface media, collected from RSV-infected cells, to mock-infected epithelium did not increase neutrophil adhesion and after 1 h we detected two to three times fewer adherent neutrophils compared to the RSV-infected epithelium (p<0.0001) (figure 2c and d).

### Neutrophil transepithelial migration during RSV infection causes epithelial cell damage and reduces ciliary beat frequency

**FIGURE 3:**
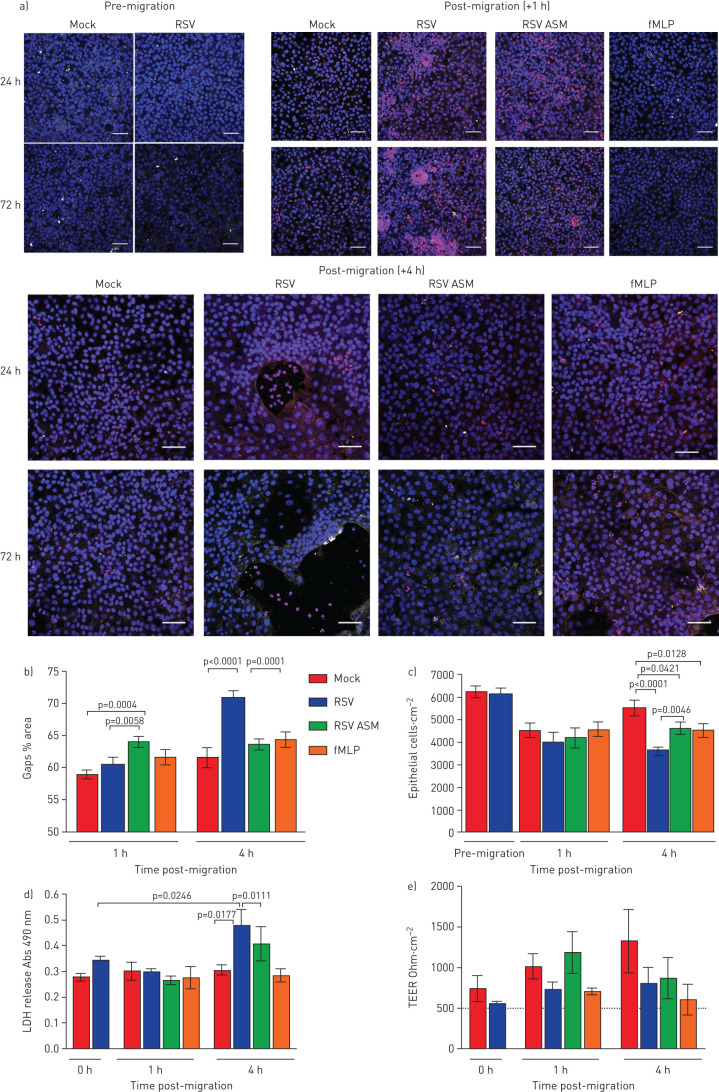
Neutrophil transepithelial migration increases the damage caused to respiratory syncytial virus (RSV)-infected ciliated epithelium. a) Representative confocal images of RSV-infected human nasal ciliated epithelial cells grown at an air–liquid interface following neutrophil (red) transepithelial migration for 1 or 4 h. Cells were stained with antibodies against acetylated tubulin to detect the ciliary microtubules (white) and nuclei were stained using Hoechst (blue). Gaps in the epithelial layers are shown by large (>500 µm^2^) black areas. Scale bars=50 μm. b) Gap analysis. c) The number of epithelial cells attached to membrane inserts after 72 h recombinant RSV infection and 1 and 4 h after neutrophil migration. Epithelial cells were quantified by counting the DAPI-stained nuclei >50 µm^2^ in area using ImageJ. Bars represent the mean±sem for five images per well, n=3 epithelial donors, n=2 heterologous blood donors and n=1 autologous blood donor. d) At 72 h post-infection, lactate dehydrogenase (LDH) release was measured in apical surface media of nasal airway epithelial cells (nAECs) post neutrophil migration for 1 and 4 h. Bars represent the mean±sem for n=4 epithelial donors, n=3 heterologous blood donors and n=1 homologous blood donors. e) Transepithelial electrical resistance (TEER) of each well as measured using a voltohmmeter. Data are presented as the mean±sem of n=4 epithelial donors, n=3 heterologous blood donors and n=1 homologous blood donor for mock-infected, RSV-infected, mock-infected and exposed to apical surface media (ASM) collected from RSV-infected cells or mock-infected and exposed apically to the chemoattractant N-formylmethionine-leucyl-phenylalanine (fMLP). Statistical significance is shown.

We did not detect any signs of epithelial damage following neutrophil transepithelial migration at 24 h post-RSV infection (supplementary figure S3). Nor did we detect any increase in makers of epithelial damage after 1 h of neutrophil migration across epithelium infected with RSV for 72 h (figure 3b). However, 4 h after neutrophil transepithelial migration across epithelium infected with RSV for 72 h, we detected large gaps (70.8±4.6% area) in the RSV-infected epithelial layer compared to the mock-infected epithelium (61.6±6.0% area) (p<0.0001) (representative images shown in figure 3a, quantitative data shown in figure 3b). These gaps were associated with epithelial rounding, as observed using time-lapse microscopy (supplementary video 2), a loss of epithelial cells (figure 3c) and an increase in LDH release (figure 3d) which was greater in RSV-infected cultures compared to the mock-infected (p<0.0001). Interestingly, this epithelial cell loss did not correspond with an increase in red dextran flux (supplementary figure S4) or decrease in TEER (figure 3e). We did not find any differences in TEER compared with the mock-infected cells exposed to either RSV-infected apical surface media or fMLP (figure 3e). To determine whether physical impediments that could alter the red dextran and electrical current outputs were compensating for the loss in epithelial cells, we observed microscopically the subepithelial layer (top of the membrane insert) over time and found that after 4 h, the basolateral neutrophils had accumulated as a sediment (supplementary figure S5).

**FIGURE 4:**
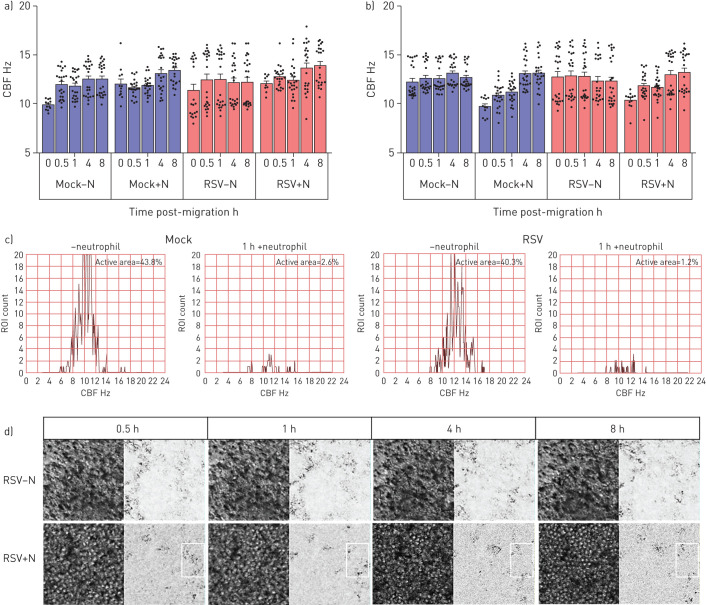
The effect of respiratory syncytial virus (RSV) and neutrophil transepithelial migration on ciliary beat frequency (CBF) of human ciliated epithelial cells infected with mock or RSV A2 for a) 24 h or b) 72 h as determined using fast Fourier transformation of high-speed video microscopy videos by ciliaFA. Bars represent the mean±sem of n=12 areas per well for cultures mock-infected, mock-infected following neutrophil transepithelial migration, RSV-infected or RSV-infected following neutrophil transepithelial migration. Statistical comparison between all groups was performed using a paired t-test. n=3 epithelial donors, n=3 heterologous blood donors. Statistical significance is shown. c) Representative histograms of the frequency distribution of CBF from 1600 regions of interest taken from a representative field of view. d) Representative image of RSV-infected (72 h post-infection) ciliated regions of interest, at 0.5, 1, 4 and 8h post neutrophil migration, plus or minus neutrophils. Phase image (left), and CiliaFA readout image (right), showing moving ciliated cells identified (black) during CiliaFA analysis.

Using high-speed video microscopy, we investigated whether neutrophil migration alters ciliary activity, which is a sensitive indicator of cell toxicity. We found that 30 min after the addition of neutrophils, the mean±sd CBF of 36 2856-µm^2^ ciliated areas of interest imaged from cultures infected with RSV for 72 h was 12.84±0.79 Hz compared to 15.06±0.95 Hz in RSV-infected cultures without neutrophils (figure 4b) (p<0.05). Although this was not statistically different, an absolute difference in CBF of 2 Hz has been shown to be clinically significant in reducing mucociliary clearance [27]. 8 h after the addition of neutrophils, the average CBF of the mock- and RSV-infected cultures appeared to recover to pre-neutrophil levels (figure 4b). However, when examined closely, comparing an individual region of interest over time, it was clear that neutrophil migration led to far fewer areas (1.4% region of interest compared to 40.3% in RSV-infected cultures without neutrophils) demonstrating active beating cilia (defined as >3 Hz) (figure 4c).

### Epithelial damage correlates with neutrophil degranulation and higher apical concentrations of neutrophil elastase

**FIGURE 5:**
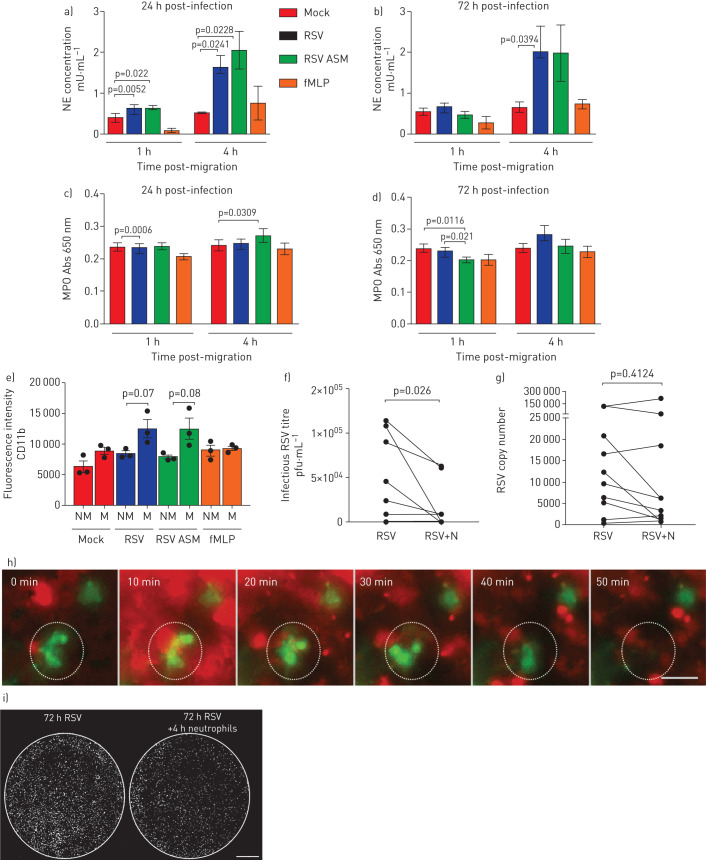
Release of neutrophil-derived products and neutrophil activation after transepithelial migration across respiratory syncytial virus (RSV)-infected ciliated epithelial cells. Levels of a,b) neutrophil elastase (NE) and c,d) myeloperoxidase (MPO) were measured in apical surface media after migration across mock- or RSV-infected (24 h/72 h infection) ciliated epithelial monolayers following neutrophil migration for 1 and 4 h. For all, bars show mean±sem of n=4 epithelial donors, n=3 heterologous blood donors and n=1 homologous blood donor. Statistical comparison between all groups was performed using a paired t-test. Statistical significance is shown. e) Cell surface expression of CD11B is increased on neutrophils that migrated across RSV-infected epithelial cells infected for 72 h show compared to nonmigrated neutrophils. The percentage of migrated (M) and nonmigrated (NM) neutrophils expressing CD11B was calculated by staining for cell surface expression of CD11b (PE) were determined by flow cytometry. Neutrophils were gated on initially using a PE-positive gate. Using this population, the geometric mean fluorescence intensity of PE fluorescence was calculated. Bars show mean±sem of n=3 epithelial donors, n=3 heterologous blood donors. Statistical significance is shown, p<0.05. f,g) Viral titre at 72 h post-infection as determined by f) plaque assay (pfu·mL^−1^) or g) quantitative reverse transcriptase-PCR of whole-well lysates showing a decrease in infectious RSV 4 h post-neutrophil migration. n=10 technical repeats from n=6 epithelial donors, n=5 heterologous blood donors and n=1 homologous blood donor. h) Time-lapse fluorescence microscopy showing elimination of green fluorescent protein-positive (green) epithelial cell within 50 min of the addition of neutrophils (red) to the basolateral side of the epithelial cells. This is also shown in video 3. Scale bar=50 μm. i) Whole-well fluorescence microscopy scan of a representative membrane insert infected with RSV for 72 h cells before (left) and 4 h after (right) neutrophil migration. Each white spot indicates an RSV-infected epithelial cell. i) Whole-well scans using fluorescence microscopy showing the effect of neutrophil migration on overall numbers of RSV-infected epithelial cells (RSV+ve cells=white).

Neutrophils release several toxic products, including myeloperoxidase (MPO) and neutrophil elastase. To determine whether the presence of these products correlated with the epithelial damage, we measured the concentration of these products in the apical surface media. We found that the concentration of neutrophil elastase, in apical surface media of the nAEC cultures, was more than three-fold greater following neutrophil migration across RSV-infected epithelium for 4 h (but not 1 h) at 72 h post-infection (figure 5a and b) with a mean±sem of 2.0±0.6 mU·mL^−1^, compared to 0.6±0.1 mU·mL^−1^ in the mock-infected cultures (p=0.039) (figure 5b). We did not find a significant difference in MPO in apical surface media from RSV-infected cultures following neutrophil migration for 1 or 4 h compared to the mock-infected cultures (p=0.0006) (figure 5c and d). Furthermore, we did not find a difference in cellular expression of MPO on neutrophils migrated across mock compared to RSV-infected epithelium (data not shown).

We found that expression of cellular neutrophil activation marker CD11b was 1.4-fold greater on neutrophils that had migrated across epithelium infected with RSV for 72 h (mean±sem fluorescence intensity of 1.2×10^4^±1.5×10^3^) compared to <8.5×10^3^±4×10^2^ nonmigrated neutrophils (p<0.05) (figure 5e), and compared to neutrophils not exposed to the epithelial cells (5.6×10^3^±1.7×10^3^) (p<0.05). In addition, we found that expression of CD11b was greater on neutrophils that had migrated across RSV-infected epithelium after 1 h compared to neutrophils that had migrated across mock-infected compared to RSV-infected epithelium (p<0.05) (figure 5e).

### Neutrophil transepithelial migration reduces the infectious viral load

Apical surface media and apical cell layers collected 4 h after migration exhibited significantly lower viral titre of 1.6×0^4 ^pfu·mL^−1^ in RSV-infected ciliated nAEC cultures after neutrophil migration, compared to 4.4×10^5 ^pfu·mL^−1^ in RSV-infected nAECs without neutrophil migration (p=0.03) (figure 5f). This is a mean±sem difference in viral titre of −2.7×10^4^±1.2×10^4 ^pfu·mL^−1^ (supplementary figure S6a). These findings were confirmed by the observation of fewer GFP-positive cells following neutrophil migration from whole-well scans (figure 5i) and time-lapse fluorescence microscopy, which showed less GFP expression following neutrophil migration (video 3 and figure 5h). There was no significant difference in viral RNA in RSV-infected cultures 4 h after neutrophil migration (figure 5g and supplementary figure S6b). Interestingly, neutrophils exposed to RSV in absence of nAECs also showed a reduction in infectious viral load after 4 h, with a difference in viral titre of −1.1×10^5^±1.3×10^4 ^pfu·mL^−1^ (supplementary figure S7a); again there was no difference in viral RNA (supplementary figure S7b).

### Blocking neutrophil β_2_-integrin LFA-1 reduces neutrophil adherence and epithelial damage

**FIGURE 6:**
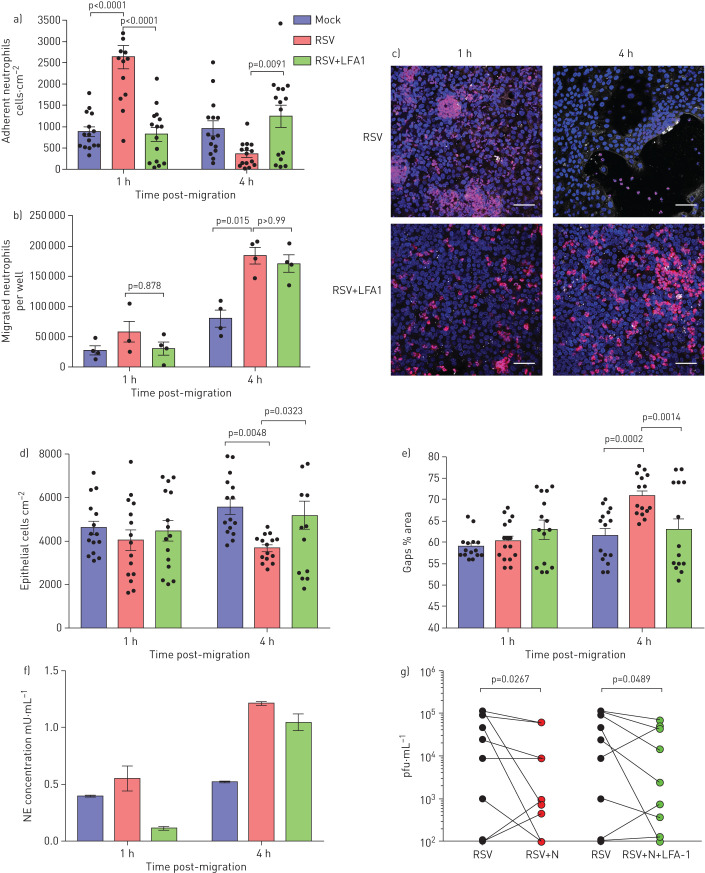
Blocking neutrophil β_2_-integrin leukocyte function-associated antigen-1F (LFA1) reduces neutrophil adherence and epithelial damage. a) Number of neutrophils attached to membrane insert 72 h post-respiratory syncytial virus (RSV) infection and post-migration for 1 and 4 h were quantified with or without addition of an LFA1 inhibitor. Neutrophils were counted using ImageJ; the average number of neutrophils from all images is shown (five images per donor, n=3 epithelial donors, n=2 heterologous blood donors and n=1 homologous blood donor). b) Numbers of neutrophils that migrated across RSV-infected ciliated cell monolayers after 24 and 72 h RSV infection were quantified with or without addition of an LFA1 inhibitor. LFA1 group is a RSV-infected membrane insert with the LFA1 blocker added to the apical surface media collected from RSV-infected cells underneath (apical). c) Representative microscopy images of ciliated epithelial monolayers infected with RSV for 24 and 72 h, stained with a nuclei stain (Hoechst) following neutrophil (red) migration for 1 and 4 h with or without addition of an LFA1 inhibitor. Scale bars=50 μm. d) Numbers of epithelial cells attached to membrane insert were quantified after neutrophil migration for 1 and 4 h in RSV-infected groups with or without addition of an LFA1 inhibitor. Epithelial cells were counted using ImageJ, the average number of epithelial cells from all images is shown (five images per donor, n=3 epithelial donors, n=2 heterologous blood donors and n=1 homologous blood donor). e) Lactate dehydrogenase (LDH) release was measured in apical surface media of airway epithelial cells post-neutrophil migration for 1 and 4 h in RSV-infected groups with or without addition of an LFA1 inhibitor. f) Neutrophil elastase (NE) was measured in apical surface media after migration across 72 h RSV-infected ciliated epithelial monolayers with and without LFA-1 inhibitor following neutrophil migration for 4 h. n=2 epithelial donors, n=1 heterologous blood donor and n=1 homologous blood donor. g) Viral titre as determined by plaque assay (pfu·mL^−1^) of whole-well lysates showing a decrease in infectious RSV 4 h post-neutrophil migration (n=9 technical repeats from n=6 epithelial donors, n=5 heterologous blood donors and n=1 homologous blood donor). Statistical significance is shown.

We found that ICAM-1 expression was significantly greater on epithelial cells after 72 h RSV infection compared with mock-infected epithelial cells (p<0.05) (supplementary figure S2) and so we focussed on this time point for these studies. We found fewer neutrophils adhered to RSV-infected epithelium when exposed to an inhibitor that blocked the interaction of neutrophil LFA-1 and ICAM-1 on epithelial cells compared to the RSV-infected epithelium alone (p<0.0001) (figure 6a). In addition, we observed smaller, less frequent neutrophil aggregates when using the LFA-1 inhibitor compared to the RSV-infected epithelium (observational data). There was no difference in the number of neutrophils that migrated across the RSV+LFA-1 group compared to the RSV group (figure 6b), suggesting that addition of the LFA-1 inhibitor does not affect neutrophil chemotaxis across the epithelium.

We found less epithelial cell shedding and fewer gaps formed in the epithelial layer after 4 h when the RSV-infected epithelium exposed to the LFA-1 inhibitor (figure 6c–e) compared to the RSV-infected epithelium (p=0.003). As before, we did not detect any changes in TEER and red dextran flux in RSV-infected epithelium exposed to the LFA-1 inhibitor (figure S6). Interestingly, we found that after 4 h neutrophil migration RSV-infected epithelium and RSV-infected epithelium exposed to the LFA-1 inhibitor both showed higher neutrophil elastase release compared to the mock-infected cells (p=0.03) (figure 6f). In addition, exposure to the LFA-1 inhibitor reduced viral titre of RSV-infected epithelial cells after neutrophil migration (p=0.049) (figure 6g).

## Discussion

We showed that neutrophil migration and adherence to RSV-infected airway epithelial cells was associated with greater epithelial cell damage, greater neutrophil degranulation and a reduction in infectious viral load. We showed that these effects are mediated, at least in part, by the β_2_-integrin ligand LFA-1 on neutrophils binding to the ICAM-1 receptor on nAECs, as inhibition of this interaction prevented neutrophil-associated epithelial damage.

The airways of infants with RSV bronchiolitis contain a large infiltrate of neutrophils which have migrated from the bloodstream and across the airway epithelium. There is limited understanding of how this influx of neutrophils contributes to the pathophysiology of RSV infection. In contrast, previous studies using *in vitro* human ciliated airway epithelial cell models of RSV infection, while reporting that viral replication within ciliated nAECs peaks in the first 24–72 h, show little evidence of damage to the epithelial cells during this period of RSV infection [6, 9]. Using this new human airway model to investigate the neutrophil-mediated response in RSV-infected ciliated epithelial airway cells and mock-infected cells, we have shown the following for the first time.

1. Infection with RSV increased the numbers of neutrophils that migrated across the epithelium, after both early and late infection. The presence of apical surface media in the absence of infected cells also achieved this effect after 72 h. This supports the clinical findings that neutrophil infiltration in the lungs of infants with RSV bronchiolitis [10] correlates with disease severity [15, 28, 29]. In addition, infection of the epithelium with RSV caused more neutrophils to remain adherent to the epithelium and not detach into the apical surface media. Comparison with mock-infected cells exposed to apical surface media from infected cells suggested that this increase in neutrophil adherence was dependent on RSV infectious particles present in or around the epithelial cells. The adherent neutrophils appeared to form large clusters, suggesting that the neutrophils coordinate or localise their migration. This is a similar finding to that of Yonker *et al.* [30] who showed that neutrophil transepithelial migration across epithelium infected with *Pseudomonas aeruginosa* led to clumping of neutrophils. Neutrophil swarming has been observed in other infections and inflammatory diseases [31–33].
2. Neutrophil transepithelial migration across differentiated primary human airway epithelium infected with RSV was associated with epithelial damage, including cell shedding and reduced CBF. We detected an immediate (0–30 min) reduction in CBF following neutrophil transepithelial migration across ciliated epithelium infected with RSV for 24 h. This sensitive readout could be an early indication of cell damage, with neutrophil transepithelial migration leading rapidly to cilia slowing down. After 4 h neutrophil transepithelial migration there was an increase in CBF of RSV-infected epithelium, back to pre-neutrophil levels. This may indicate that neutrophil migration leads to a loss of the slower beating or damaged cilia, thus explaining the greater average beat frequency of the sampling area after this duration of infection.
3. Epithelial damage was associated with greater neutrophil activation and degranulation, with greater release of neutrophil elastase into the apical surface media. Neutrophil degranulation is an important feature of the host response and pathophysiology of RSV infection [34, 35]. A previous *in vitro* study demonstrated that RSV stimulated MPO release from neutrophils [36], which is consistent with our findings. The levels of neutrophil elastase, MPO and matrix metalloproteinase-9 in sputum and bronchoalveolar lavage fluid have also been shown to correlate with indices of disease severity in airway diseases [37, 38].
4. Loss of epithelial cells and increased LDH release was specific to RSV-infected epithelium. We did not detect a change in TEER. This may be due to the nonmigrated neutrophils forming a sediment on the basolateral side of the membrane, which could increase the electrical resistance and compensate for the gaps in apical epithelial cells we detected. Despite the migration of similar numbers of neutrophils towards apical surface media collected from RSV-infected cells or the neutrophil chemoattractant fMLP, compared to migration across RSV-infected epithelium, this did not lead to epithelial cell shedding or LDH release. This suggests that damage is dependent on the presence of RSV-infected epithelial cells. Sloughing off of these infected epithelial cells may lead to obstruction in the small airways of young infants [39]. RSV NS2 protein has been shown to contribute to epithelial shedding and acute airway obstruction in an animal model [11].

Furthermore, we showed that neutrophil-mediated RSV-induced epithelial damage leads to a reduction in viral load. This antiviral activity could be directly mediated by neutrophils, either by degranulation or by phagocytosis of RSV-infected cells, thereby preventing further viral spread. Additional experiments conducted with neutrophils and RSV in absence of infected nAECs supported the findings that neutrophils have an antiviral effect in that they reduce the number of infectious particles, but not the amount of RNA (supplementary figure S7). Some previous studies support this finding and neutrophils have been shown to be beneficial in viral respiratory tract infections [40–42] and are thought to contribute to antiviral defence [43, 44]. However, studies using neutrophil-depleted mice concluded that neutrophils were unlikely to play a major role in viral clearance during RSV infection, as viral loads [45] and lung damage [40] were equivalent to those reported in the wild-type mice. We found that neutrophil transepithelial migration reduced the amount of infectious RSV, but did not alter the amount of viral RNA recovered from RSV-infected ciliated epithelium. This difference may be due to the sensitivities of the assays with inactivated viral particles present in neutrophils and/or epithelial cells that have intact or partially fragmented viral RNA.

Since we observed less epithelial damage in conditions where fewer neutrophils adhered, we hypothesised that the neutrophil adherence to the RSV-infected epithelium was responsible for the epithelial damage. To investigate this, we used an inhibitor that blocked the interaction between LFA-1 on neutrophils and ICAM-1 on nAECs. This inhibitor reduced neutrophil adherence to RSV-infected epithelium and considerably reduced epithelial damage, although the number of migrated neutrophils and neutrophil elastase release was unaffected. This suggests that the epithelial damage is mediated by the proximity or direct interaction of neutrophils with RSV-infected epithelium. The organisation and clustering of adherent neutrophils could be an important mechanism that drives RSV disease severity. Previous reports have shown that localised leukotriene B4 signalling is responsible for neutrophil swarming and the coordinated clustering of neutrophils could accelerate neutrophilic inflammation [33]. The accessibility to the cells of interest in our model offers a methodological advantage over *in vivo* and intravital methods and this could allow us to investigate human cellular and transcriptomic changes at a single cell level. Future work by our group aims to compare the transcriptomic and functional behaviour of neutrophils during RSV infection.

In conclusion, this study reveals that neutrophil transepithelial migration and adherence to epithelial cells quickly results in poorer ciliary function, tissue damage and increased viral killing in RSV-infected ciliated cell cultures. Further work to investigate the mechanisms of migration, including neutrophil dynamics, signalling, swarming and activation, could improve our understanding of the infant immune response in RSV bronchiolitis and help develop new therapeutics.

## Supplementary material

10.1183/13993003.02216-2019.Supp1 **Please note:** supplementary material is not edited by the Editorial Office, and is uploaded as it has been supplied by the author.
Supplementary methods erj-02216-2019.methods
Supplementary figures erj-02216-2019.figures
Supplementary statistical information erj-02216-2019.stats
Supplementary video 1 erj-02216-2019.Video_1
Supplementary video 2 erj-02216-2019.Video_2
Supplementary video 3 erj-02216-2019.Video_3

## Shareable PDF

10.1183/13993003.02216-2019.Shareable1 This one-page PDF can be shared freely online.
Shareable PDF ERJ-02216-2019.Shareable

## Supplementary Material

ERJ-02216-2019.Shareable.pdf
