# Supplementary material for: β2-integrin LFA1 mediates airway damage following neutrophil transepithelial migration during respiratory syncytial virus infection
Source: Eur Respir J. 2020 Aug 6;56(2):1902216. doi: 10.1183/13993003.02216-2019 (PMC7406857; doi:10.1183/13993003.02216-2019)
Supplement: Supplementary file 1 [file erj-02216-2019.methods.pdf]

TITLE: β2 integrin LFA1 mediates airway damage following neutrophil trans-epithelial migration during RSV infection. METHODS

AUTHORS AND AFFILIATIONS:
Jenny A. Herbert[1], Yu Deng[1,2], Elisabeth Robinson[1], Rosalind L. Smyth[1], Claire M. Smith[1]
[1] Respiratory, Critical Care & Anaesthesia, Great Ormond Street Institute of Child Health, University College London (UCL), London, United Kingdom.

Corresponding author:
Claire Smith
c.m.smith@ucl.ac.uk
Tel: +44 (0)20 7905 2183

Email address of co-authors:
Jenny Herbert (Jenny.Herbert@ucl.ac.uk)
Yu Deng (y.deng@ucl.ac.uk)
Elisabeth Robinson (elisabeth.robinson.17@ucl.ac.uk)
Rosalind Smyth (rosalind.smyth@ucl.ac.uk)

A Video Methodology can be found here: https://youtu.be/NRFRuriSWks

PROTOCOL:

1. Isolation of primary AECs from nasal brushings

1.1. Collect airway epithelial cells by performing a nasal brushing on a healthy adult donor as described elsewhere[14]. Commercially available nasal epithelial cells (i.e. from Lonza or Epithelix Sarl) can also be used. The methods shown here can be used for cells obtained from both nasal brushings and commercially available human nasal epithelial cells.

1.2. Process nasal brushings cells immediately (i.e. go to step 1.3) or store in the fridge overnight in 2 ml M199 media supplemented with 2.5μg/ml amphotericin B, 100 U/ml penicillin and 100 μg/ml streptomycin.

1.3. Collect the nasal epithelial cells by centrifugation at 600xg.

1.4. Resuspend in F-media as is described elsewhere [15] (F-media composition: Dulbecco's modified Eagle's medium (DMEM) and F12 at a 3:1 ratio with 100 U/ml penicillin and 100μg/ml streptomycin and 7.5% (v/v) fetal bovine serum supplemented with 5 mM Y-27632, hydrocortisone (25 ng/ml), epidermal growth factor (0.125 ng/ml), insulin (5 mg/ml), 0.1 nM cholera toxin, amphotericin B (2.50 μg/ml)).

1.5. Cells are ready to be seeded as described in section 2.2

2. Expansion of primary AECs using co-culture with 3T3-J2 feeder cell layers

All steps should be performed in a sterile environment using a Class II safety cabinet.

2.1. Prepare 3T3-J2 feeder layers as described previously [15]. Briefly:

2.1.1. Culture 3T3-J2 mouse embryonic fibroblast in DMEM supplemented with 10% (v/v) calf serum, 100 U/ml penicillin and 100 μg/ml streptomycin until 70-80 % confluent.

2.1.2. Add 150 μl of stock mitomycin (0.4 μg/ml in PBS) to a T175cm^2 flask of 3T3-J2 cells with 15 ml fresh media to mitotically inactivate cells.

2.1.3. Incubate for 2 hours at 37°C 5 % CO_2.

2.1.4. After incubation, remove all media by aspiration.

2.1.5. Rinse 3T3-J2 cells with sterile PBS.

2.1.6. Dissociate 3T3-J2 cells using 1x trypsin. Incubate flasks at 37°C 5 % CO_2 for roughly 5 minutes or until >95% cells are detached.

2.1.7. Gently tap the T75cm^2 flasks to promote 3T3-J2 cellular detachment.

2.1.8. Collect these cells in fresh PBS to inactivate trypsin and centrifuge at 200xg for 3 minutes.

2.1.9. Resuspend the cell pellets in DMEM and count the number of viable cells using a haemocytometer and trypan blue exclusion.

2.1.10. Seed cells at a density of $1x10^6$ / T75cm^2 flask in DMEM media and incubate at 37°C 5 % CO_2.

2.1.11. 3T3-J2 feeder cells must be used within 48 hours of cell seeing. One confluent T175cm^2 flask of cells should make between 3-6 feeder flasks.

2.2. Seed primary AECs into feeder layers at a density of $5x10^5$/ flask. Seed cells directly into one or two 3T3-J2 feeder layers for propagation. A vial of commercial frozen AECs can be split into two 3T3-J2 feeder flasks. AECs should not be used beyond passage 5 as they

will not differentiate.

2.3. AECs are ready for use once the flasks are nearly confluent. To separate the 3T3-J2 fibroblasts from the basal cells expose them to differential concentrations of trypsin. 3T3-J2 are more sensitive to trypsin so should dissociate first while the AECs remain attached for a longer period. To do this:

2.3.1. Aspirate media from T75cm^2 flasks and rinse cells in sterile PBS.

2.3.2. Add 2ml of 1x Trypsin/ EDTA to each flask. Make up a 1x working trypsin solution by diluting the 10X stock in sterile PBS.

2.3.3. Incubate flasks at 37°C 5 % CO_2 for roughly 5 minutes or until >95% fibroblasts are detached

2.3.4. Gently tap the T75cm^2 flasks to promote 3T3-J2 cellular detachment, the AECs should remain attached.

2.3.5. Rinse flasks in PBS to inactivate trypsin and remove residual fibroblasts.

2.3.6. Detach and dissociate the remaining AECs. Add 2ml of trypsin and incubating for 5-10-minutes at 37°C 5 % CO_2.

2.3.7. Collect these cells in fresh F-media and centrifuge at 200xg for 3 minutes.

2.3.8. Resuspend the cell pellets in F-media and count the number of viable cells using a Neubauer haemocytometer and trypan blue exclusion.

3. Collagen coating permeable membrane inserts.

All steps should be performed in a sterile environment using a Class II safety cabinet.

3.1 Make collagen coating solution: dilute 3 mg/ml type I collagen stock 1:100 in sterile PBS (30 μg/ml working stock).

3.2 Remove a 24 well polyethylene terepthalate (PET) membrane insert with 3 μm pore size from sterile packaging and place in a new 24 well plate.

3.3 Coat membrane insert on both sides of the membrane.

3.3.1 Add 100 μl of collagen working solution to the top of all membrane insert.

3.3.2 Invert plate and remove plate bottom (membrane insert are now standing upright on the plate lid). The collagen solution below the membrane insert membrane remains in place by surface tensions. Ensure the solution does not drip down the side of the membrane insert.

3.3.3 Add 100 μl of collagen working solution to the other side of the membrane insert.

3.3.4 Replace the 24 wells plate to cover the membrane insert ensuring not to disturb the collagen solution.

3.3.5 Incubate the membrane insert at room temperature for at least one hour.

3.4 After incubation aspirate collagen solution from both sides of the membrane insert membrane.

3.5 Rinse membrane insert with sterile water on both side of membrane and return to 24 well plate.

3.6 Leave lid off plate and allow membrane insert to dry in the tissue culture hood for 1 hour.

3.7 Replace lid and seal plates using parafilm. Store plates at room temperature for up to a month.

4. Seeding AECs onto permeable membrane inserts.

All steps should be performed in a sterile environment using a Class II safety cabinet.

4.1. Invert a collagen coated 24 well membrane insert in a sterile 12 well plate (one membrane insert/ well).

4.2. Seed AECs onto the bottom of the 3μm pore membrane insert membrane at a density of 300,000/cm^2 in 70 μl of F-media. Place the lid over the plate and incubate at 37°C 5 % CO_2 for 4-6 hours. The 3μm membrane insert from Greiner have 0.6 x 10^6 /cm^2 pores, this is a lower density than the other commercially available from Greiner and the Corning (2 x 10^6 /cm^2). We found that the higher pore density membrane inserts impair the ability to visualize cells on the membrane and to measure ciliary beat frequency.

4.3. After incubation invert the membrane inserts into a 24 well plate.

4.4. Aspirate any media on the membrane inserts.

4.5. Add 500 μl of fresh F-media underneath the membrane inserts.

4.6. Add 100 μl of F-media supplemented with 30 μg/ml Collagen I and 5 % (v/v) Matrigel on the top of the membrane inserts.

4.7. Incubate cells for 24-48 hours at 37°C 5 % CO_2.

4.8. After this period, aspirate media from both sides of the membrane insert membrane and feed cells with 100 μl of ALI media (ratio of 1:1 DMEM:Airway epithelial cell growth media) all supplements added, further supplemented with 2.5 μg/ml amphotericin B, 100 U/ml penicillin, 100 μg/ml streptomycin and 1 μm retinoic acid. Make up fresh retinoic acid as a 100 mM stock in 100 % ethanol. Cells are now at air-liquid interface (ALI).

4.9. Feed cells basolaterally every 1-2 days with fresh media,

4.10. Incubate at 37°C 5 % CO_2 in a high humidity incubator for 28 days to allow cellular differentiation.

4.10.1. Place trays of sterile water containing disinfectant to increase humidity and prevent bacterial/ fungal growth inside a tissue culture (TC) incubator.

4.11. Prepare a control membrane insert by seeding cells onto the top of a 0.4 μm or 3 μm pore insert membrane at a density of 150,000/ cm^2. Feed these cultures basolaterally with 350 μl of ALI media every 1-2 days.

4.12. Wash off any AECs that detach from the membrane during culture by adding 400 μl of ALI media apically. Aspirate this media and discard.

4.13. Primary AECs should be used after 4 weeks of ALI culture, when motile cilia are observed under an inverted microscope equipped with a S Plan Fluor ELWD 20x ADM objective with working distance of (8.2 – 6.9mm) and numerical aperture 0.45 (Nikon) and an ORCA Flash 4.0 digital camera (Hamamatsu). Our images were acquired at a resolution of 0.22um/pixel.

5. Preparation of virus for epithelial cell infection

5.1. Viral stocks preparation and quantification of viral titre is performed as described previously [12]. Propagate rgRSV expressing GFP in HEp-2 cells as described previously [4].

6. RSV infection of differentiated AECs

All steps should be performed in a sterile environment using a Class II safety cabinet.

6.1. Prior to infection, rinse the apical side of the cells with 400μl ALI media to remove any detached epithelial cells.

6.2. Measure trans-epithelial electrical resistance (TEER) using an epithelial voltohmeter to confirm cell integrity. Only cultures that give reading of >200Ω/cm^2 should be used.

6.3. Aspirate basolateral and apical media is from both sides of the membrane insert.

6.4. Remove membrane insert from the 24 well plate and place upside down in a fresh 12 well plate.

6.5. Dilute your virus stock in ALI media to give a multiplicity of infection (MOI) of 5 in 25 μl. To calculate the amount of virus required for infection based the calculation on their being roughly 1x 10^5 AECs present on the membrane insert membrane (a large number of cells dissociate during incubation for 4 weeks).

6.6. Pipette 25 μl of virus preparation on to the cell-side of each membrane insert, being careful not to touch the AECs. Note: place 25 μl of ALI media only onto the mock infected membrane inserts.

6.7. Replace the plate lid (ensuring the liquid does not touch the lid) and incubate membrane inserts at 37°C 5 % CO_2 for 1 hour. After incubation, wash cells in PBS to remove any excess liquid on the top of membrane inserts.

6.8. Place the membrane inserts in a fresh 24 well plate and add 100 μl of ALI media to the basolateral side of the cells. The apical side of membrane insert remains at ALI. Incubate at 37°C, 5 % CO_2 for 24-72 hours to allow progression of viral infection.

7. Isolation of neutrophils from whole blood

7.1. Isolate neutrophils from the whole blood of healthy adult donors or different patient groups.

7.2. Collect whole blood in 9 ml K3 EDTA Vacuette tubes, 21G needle and vacutainer adaptor (other volume tubes can be used).

7.3. Remove 1ml of blood and place in 1.5ml microfuge tube. Leave at room temperature with lid open for 20 minutes to form a buffy coat. Carefully close lid and centrifuge at 2000xg for 10 minutes. Collect the top layer and place in new microfuge tube. Store on ice until needed.

7.4. Purify neutrophils using a direct human neutrophil isolation kit (STEMCELL), as per the manufacturer’s instructions.

7.5. Resuspend neutrophils in HBSS- (calcium/ magnesium negative).

7.6. Neutrophils can be stained if required for the transmigration assay. To do this:

7.6.1. Incubate roughly 1x10^7 neutrophils with 1 μl of stock dye (calcein red orange dye (6.25 μg/μl in DMSO))

7.6.2. Incubate for 30 minutes at room temperature in the dark.

7.6.3. Wash cells in HBSS- and resuspend in HBSS+ (calcium/ magnesium positive).

7.7. Count cells with a Neubauer haemocytometer. Neutrophils are then ready to be added to the basolateral side of the membrane insert for migration.

8. Preparation of differentiated AECs for neutrophil migration assay

All steps should be performed in a sterile environment using a Class II safety cabinet.

8.1. On the morning of the neutrophil migration assay, place 600 μl of ALI media under the membrane insert for 30 minutes to allow collection of chemokines/ cytokines produced from RSV/ mock infected AECs.

8.2. Collect and pool the original apical supernatant from each test condition. Groups used in the assay include Mock, RSV infected controls.

8.2.1. Centrifuge at 1000xg for 3 minutes and discard the pellet containing any detached epithelial cells.

8.3. Place the membrane inserts into new ultra-low binding 24 well plates. Neutrophils do not adhere to the surface of these plates so can be collected post-transmigration.

8.4. Replace 400 μl of the supernatant underneath the corresponding test condition. Aliquot and store any remaining supernatant at -20°C for future analysis.

8.5. For a positive control, add 400 μl of 100nm fMLP (4 mg/ml stock in DMSO) in HBSS+ to the underside of a mock infected membrane insert.

8.6. Collect basolateral supernatants from each insert, aliquot and store at -20°C for future studies.

8.7. Return the plate to the incubator until the neutrophils are ready.

9. Neutrophil transmigration assay

All steps should be performed in a sterile environment using a Class II safety cabine

10.1. Add $5x10^5$ neutrophils in HBSS+ containing 1 % (v/v) autologous serum (collected from the whole blood see 8.3) to the basolateral side of each membrane insert.

10.2. Incubate plates at 37°C, 5 % CO_2 for 1-4 hours. Different time points can be used depending on the experiment.

10.3. After migration, collect neutrophils from the apical side of the epithelial cells (these are now referred to as the migrated neutrophils) and basolateral side (the non-migrated neutrophils) in a microfuge tube.

10.4. If the neutrophils were pre-stained, quantify the number migrated using a plate reader and compare to a standard curve of known numbers of stained neutrophils, as described previously [12].

10.5. Collect supernatants from the basolateral and apical side of the membrane insert; aliquot and store at -20°C for future analysis.

10.6. Fix membrane insert in 1 % (v/v) PFA and stain for neutrophil or epithelial cell markers of interest.

10 Analysis of ciliary function during neutrophil transmigration

10.1 To measure ciliary beat frequency, place membrane inserts a 24 well plate in an incubation chamber at 37°C and 5% CO_2 attached to an inverted microscope system.

10.2 Equilibrate cells for at least 30 minutes before image capture.

10.3 Record beating cilia using a digital video camera at a rate of 200 frames per second and capture at least 512 frames. The video area (512 x 512 pixels) should contain at least 10 ciliated cells. For each condition, at least 5 areas of the membrane insert should be

videoed for CBF analysis.

10.4 Save files as an .AVI and calculate CBF by fast Fourier transformation using open-sourced ciliaFA software as previously described [16].

REFERENCES:

1. Smith, C. M. *et al.* Ciliary dyskinesia is an early feature of respiratory syncytial virus infection. *Eur Respir J* **43,** 485–496 (2014).

2. McNamara, P. S., Ritson, P., Selby, A., Hart, C. A. & Smyth, R. L. Bronchoalveolar lavage cellularity in infants with severe respiratory syncytial virus bronchiolitis. *Arch Dis Child* **88,** 922–926 (2003).

3. Sacco, R. E., Durbin, R. K. & Durbin, J. E. Animal models of respiratory syncytial virus infection and disease. *Curr Opin Virol* **13,** 117–122 (2015).

4. Zhang, L., Peeples, M. E., Boucher, R. C., Collins, P. L. & Pickles, R. J. Respiratory syncytial virus infection of human airway epithelial cells is polarized, specific to ciliated cells, and without obvious cytopathology. *J Virol* **76,** 5654–5666 (2002).

5. Villenave, R. *et al.* In vitro modeling of respiratory syncytial virus infection of pediatric bronchial epithelium, the primary target of infection in vivo. *Proc Natl Acad Sci U S A* **109,** 5040–5045 (2012).

6. Tristram, D. A., Hicks, W. & Hard, R. Respiratory syncytial virus and human bronchial epithelium. *Arch Otolaryngol Head Neck Surg* **124,** 777–783 (1998).

7. Villenave, R. *et al.* Differential cytopathogenesis of respiratory syncytial virus prototypic and clinical isolates in primary pediatric bronchial epithelial cells. *Virol J* **8,** 43 (2011).

8. Persson, B. D., Jaffe, A. B., Fearns, R. & Danahay, H. Respiratory syncytial virus can infect basal cells and alter human airway epithelial differentiation. *PLoS ONE* **9,** e102368 (2014).

9. Weppler, A. & Issekutz, A. C. Alveolar epithelium down-modulates endotoxin-but not tumor necrosis factor alpha-induced activation of endothelium and selectively inhibits neutrophil transendothelial migration. *Exp Lung Res* **34,** 425–453 (2008).

10. Weppler, A., Rowter, D., Hermanns, I., Kirkpatrick, C. J. & Issekutz, A. C. Modulation of endotoxin-induced neutrophil transendothelial migration by alveolar epithelium in a defined bilayer model. *Exp Lung Res* **32,** 455–482 (2006).

11. Hurley, B. P., Williams, N. L. & McCormick, B. A. Involvement of phospholipase A2 in Pseudomonas aeruginosa-mediated PMN transepithelial migration. *Am J Physiol Lung Cell Mol Physiol* **290,** L703–L709 (2006).

12. Deng, Y., Herbert, J. A., Smith, C. M. & Smyth, R. L. An in vitro transepithelial migration assay to evaluate the role of neutrophils in Respiratory Syncytial Virus (RSV) induced epithelial damage. *Sci Rep* **8,** 6777 (2018).

13. Yonker, L. M. *et al.* Development of a Primary Human Co-Culture Model of Inflamed Airway Mucosa. *Sci Rep* **7,** 8182 (2017).

14. Chilvers, M. A. & O'Callaghan, C. Analysis of ciliary beat pattern and beat frequency using digital high speed imaging: comparison with the photomultiplier and photodiode methods. *Thorax* **55,** 314–317 (2000).

15. Butler, C. R. *et al.* Rapid expansion of human epithelial stem cells suitable for airway tissue engineering. *Am J Respir Crit Care Med* **194,** 156–168 (2016).

16. Smith, C. M. *et al.* ciliaFA: a research tool for automated, high-throughput measurement of ciliary beat frequency using freely available software. *Cilia* **1,** 14 (2012).

17. Hoonhorst, S. J. M. *et al.* Increased activation of blood neutrophils after cigarette smoking in young individuals susceptible to COPD. *Respir Res* **15,** 121 (2014).

18. Brockbank, S., Downey, D., Elborn, J. S. & Ennis, M. Effect of cystic fibrosis exacerbations on neutrophil function. *Int Immunopharmacol* **5,** 601–608 (2005).
