# Supplementary material for: β2-integrin LFA1 mediates airway damage following neutrophil transepithelial migration during respiratory syncytial virus infection
Source: Eur Respir J. 2020 Aug 6;56(2):1902216. doi: 10.1183/13993003.02216-2019 (PMC7406857; doi:10.1183/13993003.02216-2019)

## Supplementary Figures

**Figure S1 – Quality control of primary differentiated airway epithelial cells cultures grown from commercially source (Epithelix) or from a nasal brushing on 3 µm pore membrane inserts.** A- Trans-epithelial electrical resistance (TEER) was measured on cultures at day 28 post-ALI. B- Ciliary beat frequency (Hz) of beating cilia as measured after 28 days post-ALI. C - Airway epithelial cells stained by immunofluorescence with a marker of epithelial tight junctions (ZO-1; green), cilia (beta tubulin; red), and 49,6-diamidino-2-phenylindole (DAPI; blue) after 28 days of culture (scale bar, 50 µm).

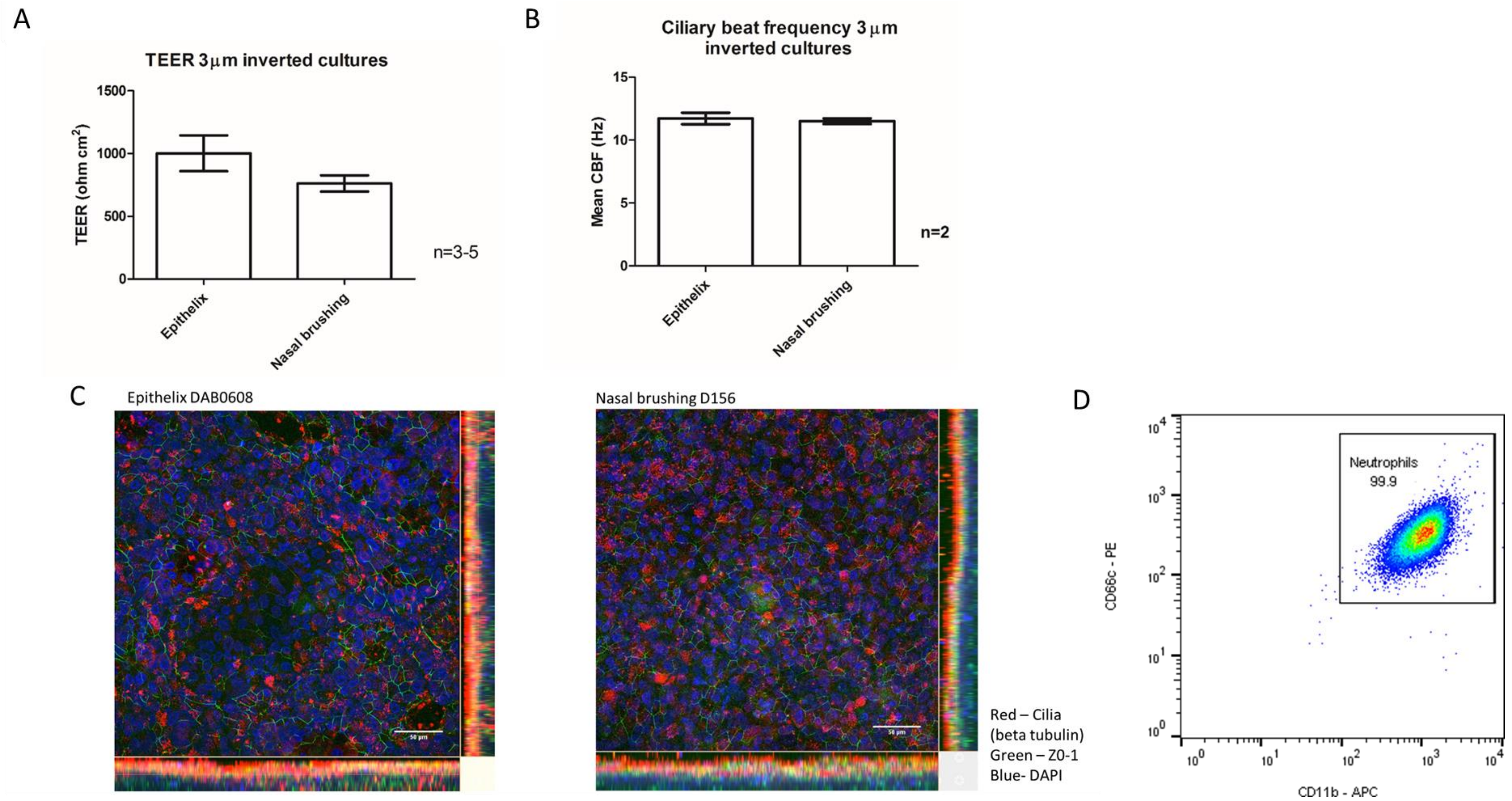
A
TEER 3μm inverted cultures
TEER (ohm cm2)
1500
1000
500
0
Epithelix
Nasal brushing
n=3-5
B
Ciliary beat frequency 3μm inverted cultures
Mean CBF (Hz)
15
10
5
0
Epithelix
Nasal brushing
n=2
C
Epithelix DAB0608
Nasal brushing D156
Red – Cilia (beta tubulin)
Green – Z0-1
Blue- DAPI
D
Neutrophils
99.9
CD66c - PE
CD11b - APC

**Figure S2- Viral replication, damage and inflammatory response of ciliated epithelial cells during RSV infection. A -** Airway epithelial cells infected with mock (left panels) or RSV-GFP (right panels) after 28 days of culture on 3 µm pore membrane. Cells were visualized on fluorescence microscope 24h (top panels) or 72h (bottom panels) post infection. The recombinant RSV we used expresses GFP when replicating in AECs and therefore infected cells can be visualized without fixation and antibody staining (scale bar, 50 µm). (**B**) Lactate dehydrogenase (LDH) release from apical supernatant of cultures infected with mock control or RSV for 24h or 72h. (**C**) TEER was measured using a voltohmmeter, (**D**) Passive movement from basolateral to apical compartments was quantified using red-dextran leakage. (**E**) IL-8 (**F**) CXCL10 (**G**) Western blot of proteins associated with tight junction formation. Statistical comparison between all groups was performed using a paired t-test. For all bars show mean ± SEM of n = 3-5 biological repeats unless otherwise stated. Statistical significance is shown. **(H)** ICAM1 expression on ciliated epithelial cells when infected with mock control or RSV for 24h or 72h as measured by fluorescence intensity. The mean (±SEM) fluorescence intensity from all images is shown (5 images per donor, 3 donors). We found that no significant difference in ICAM-1 expression at 24h post RSV infection compared to the Mock group. However, after 72h RSV infection there was significant increase in ICAM1 expression compared to the mock infected AECs. (**I**) Representative microscopy images of ciliated epithelial monolayers RSV infected for 24 and 72 hours, stained with ICAM1 (red) and nuclei stain (blue) are shown. Statistical comparison between all groups was performed using a paired t-test. For all bars show mean ± SEM of n = 3-4 biological repeats unless otherwise stated. Statistical significance is shown.

**A**

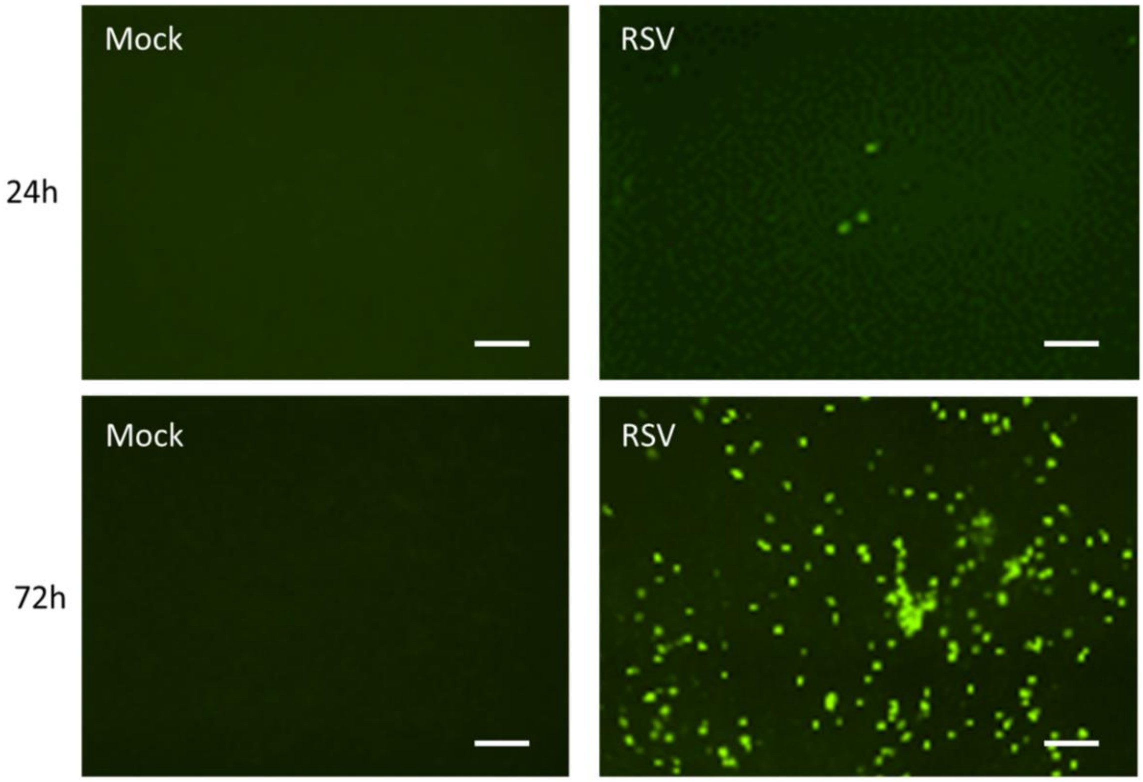

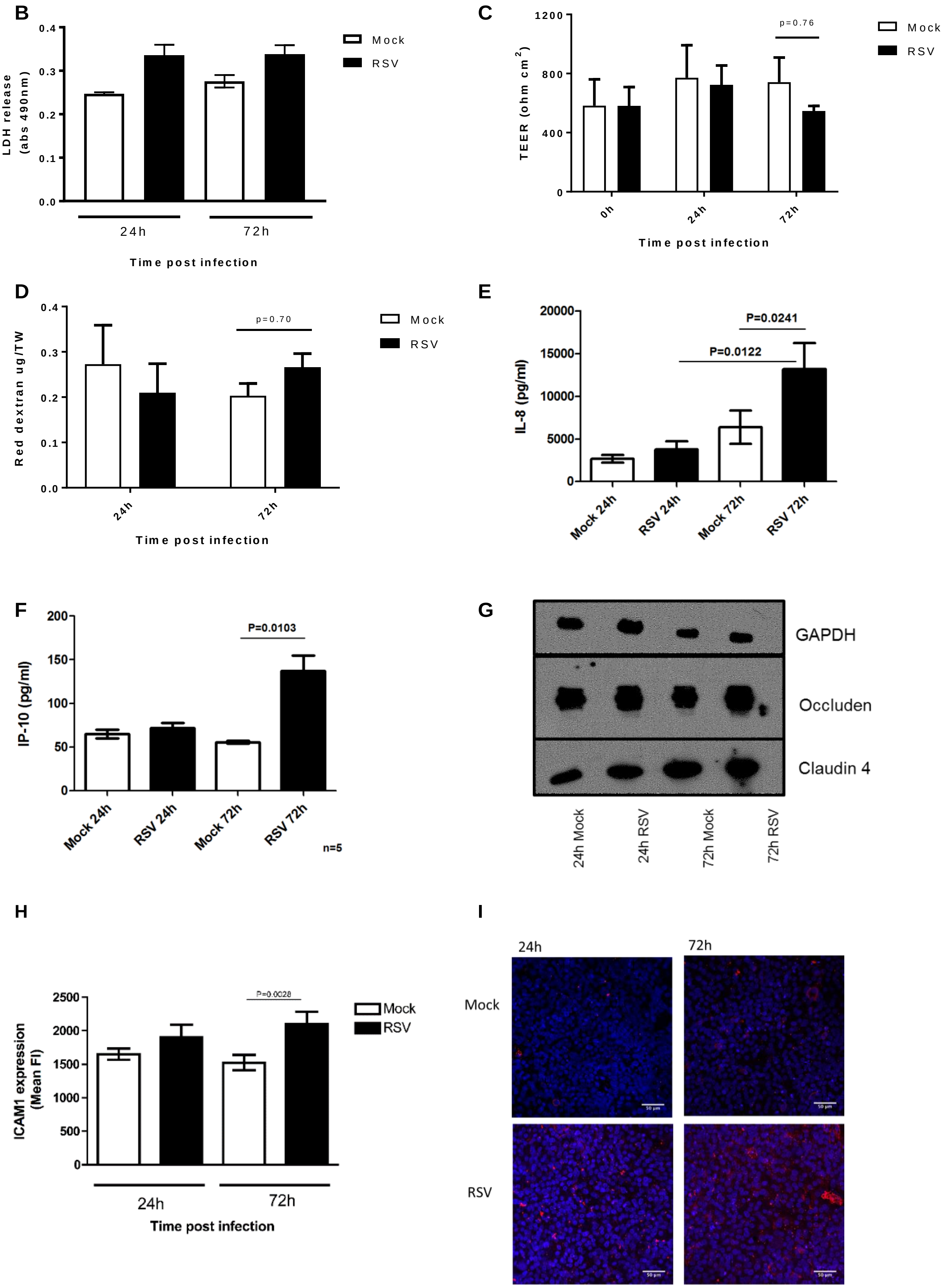
B
LDH release
(abs 490nm)
0.0
0.1
0.2
0.3
0.4
24h
72h
Time post infection
Mock
RSV
C
TEER (ohm cm²)
0
400
800
1200
p=0.76
0h
24h
72h
Time post infection
Mock
RSV
D
Red dextran ug/TW
0.0
0.1
0.2
0.3
0.4
p=0.70
24h
72h
Time post infection
Mock
RSV
E
IL-8 (pg/ml)
0
5000
10000
15000
20000
P=0.0241
P=0.0122
Mock 24h
RSV 24h
Mock 72h
RSV 72h
F
IP-10 (pg/ml)
0
50
100
150
200
P=0.0103
Mock 24h
RSV 24h
Mock 72h
RSV 72h
n=5
G
GAPDH
Occluden
Claudin 4
24h Mock
24h RSV
72h Mock
72h RSV
H
ICAM1 expression
(Mean FI)
0
500
1000
1500
2000
2500
P=0.0028
Mock
RSV
24h
72h
Time post infection
I
24h
72h
Mock
RSV

**Figure S3. Damage caused by neutrophil trans-epithelial migration through ciliated epithelium infected with RSV for 24h (A)** Gap analysis of areas with no epithelial DAPI staining (**B**) The number of epithelial cells attached to membrane inserts after 24h (F) or 72h (G) infection and 1 and 4 hours after neutrophil migration. Epithelial cells were quantified by counting the DAPI stained nuclei >50µm$^2$ in area using ImageJ. (**C**) Trans-epithelial electrical resistance (TEER) of each well as measured using a voltohmmeter. (**D**) Lactate dehydrogenase (LDH) release was measured in apical surface media of nAECs post neutrophil migration. Bars represent the mean ± SEM for cultures mock infected (white bars), RSV infected (black bars), mock infected and exposed to RSV apical surface media (checkered bars) or mock infected and exposed apically to the chemoattractant fMLP (striped bars). Statistical comparison between all groups was performed using a paired t-test. For all bars show mean ± SEM (n=4 epithelial donors, 3 heterologous and 1 homologous blood donors). * represents a significant different between the matched 1h and 4h migration time points.

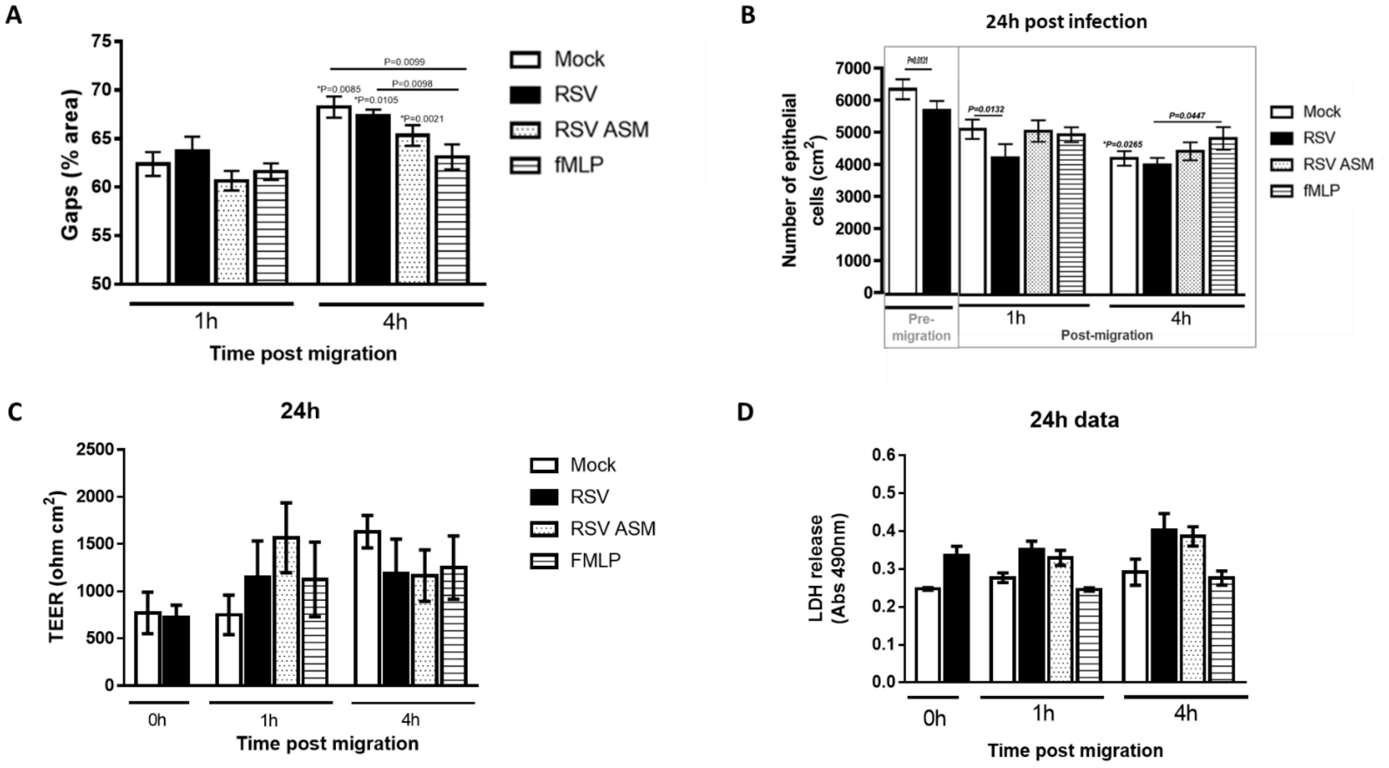

**Figure S4 Damage caused by neutrophil trans-epithelial migration through RSV infected ciliated epithelium** (**A/B**) Red-dextran (ug/well) flux from basal to apical compartments was quantified after 1 and 4 hours post neutrophil migration. Bars represent the mean ± SEM for cultures mock infected (white bars), RSV infected (black bars), mock infected and exposed to RSV apical surface media (checkered bars) or mock infected and exposed apically to the chemoattractant fMLP (striped bars). Statistical comparison between all groups was performed using a paired t-test. For all bars show mean ± SEM of n = 3-4 biological repeats unless otherwise stated. Statistical significance is shown.

A

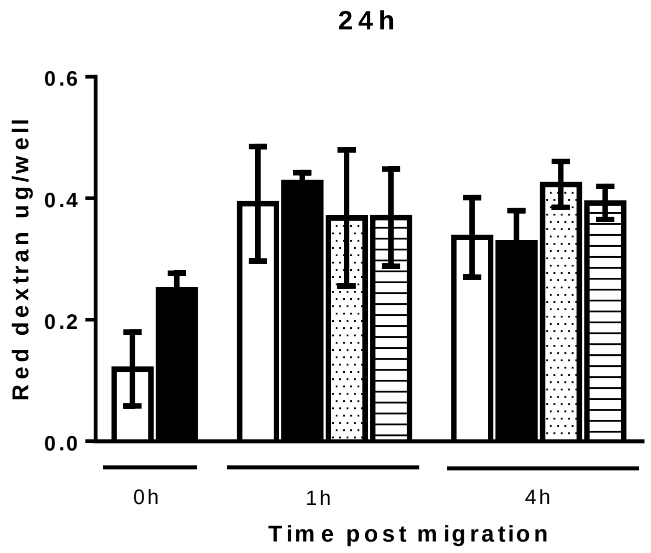


B

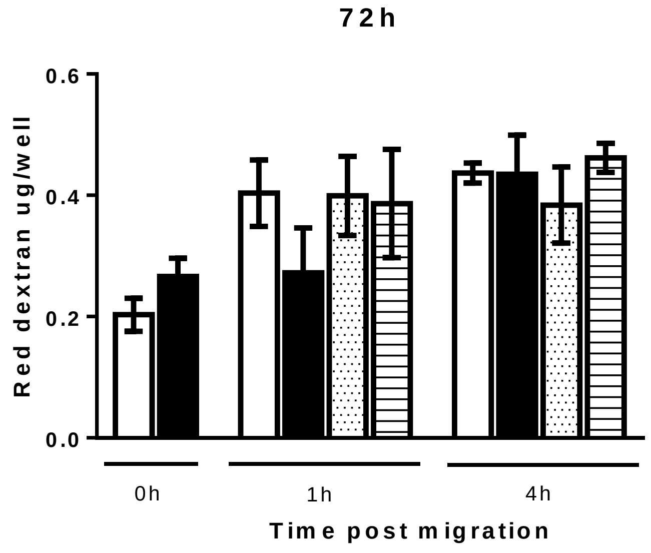

**Figure S5 The accumulation of human neutrophils on the basolateral side of respiratory syncytial virus (RSV)-infected human nasal ciliated epithelial cells grown at an air–liquid interface.** Images show the same area captured by live-confocal imaging of a 10um Z-stack (4 steps x 2.5um interval) on the basolateral side (top) of the 3um membrane insert following the addition of $5x10^5$ calcein red-orange stained neutrophils (red) for up to 4 hours. Scale bar represents 100um.

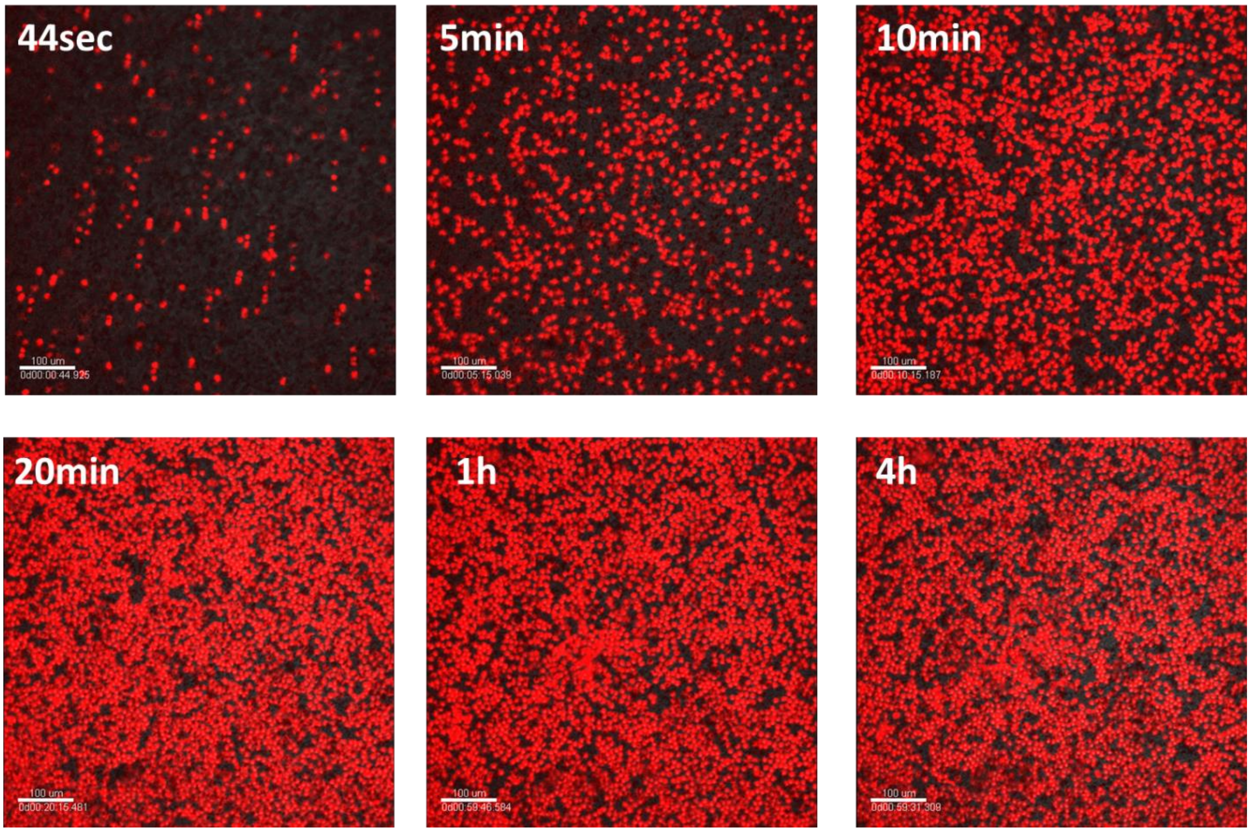

**Figure S6** The difference in RSV viral titre between infected nAEC cultures after 4h with and without neutrophils as determined by (**A**) by plaque assay and (**B**) N gene copy number by qPCR**.** Data represents difference in counts from homolgous RSV infected nAECs in absence of neutrophils.

**A**

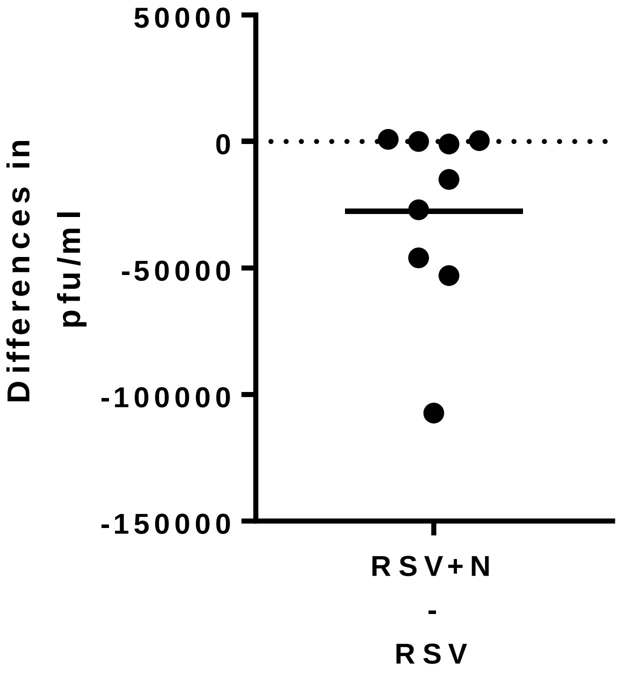


**B**

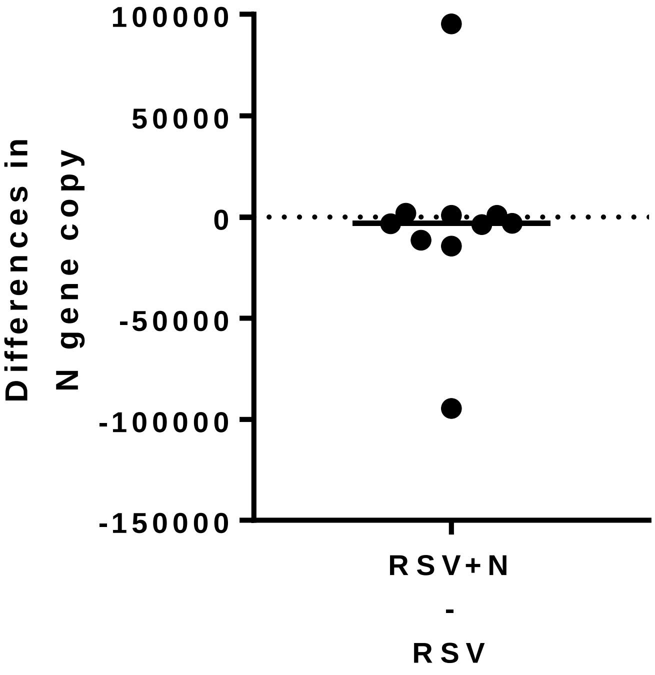

**Figure S7** The effect of neutrophils alone (no AECs) on RSV viral titre determined by (**A**) by plaque assay and (**B**) N gene copy number by qPCR (n=5 neutrophil donors, 1-2 technical repeats, all data shown) after 4h in presence of neutrophils.

**A**

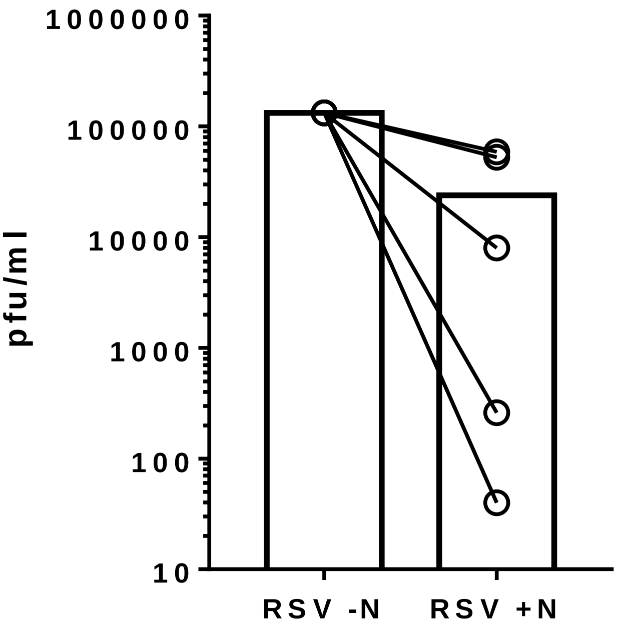


**B**

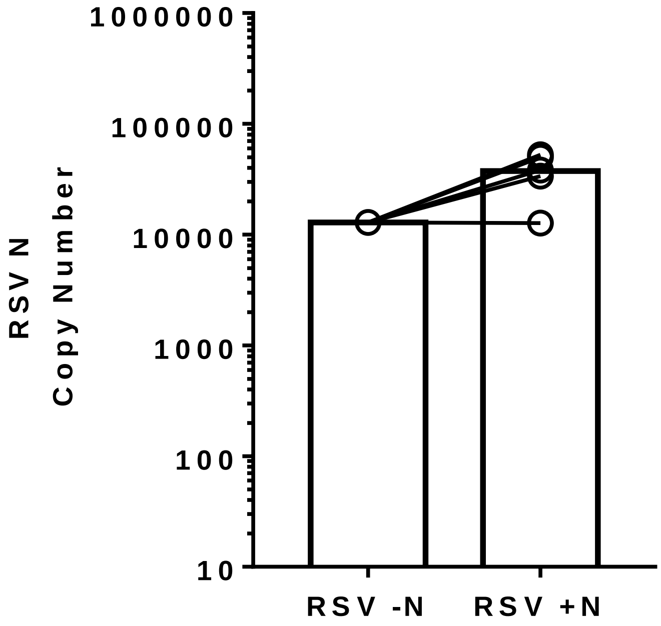

Supplement: Supplementary file 2 [file erj-02216-2019.figures.pdf]
