# Supplementary material for: β2-integrin LFA1 mediates airway damage following neutrophil transepithelial migration during respiratory syncytial virus infection
Source: Eur Respir J. 2020 Aug 6;56(2):1902216. doi: 10.1183/13993003.02216-2019 (PMC7406857; doi:10.1183/13993003.02216-2019)
Supplement: Supplementary file 3 [file erj-02216-2019.stats.pdf]

Appendix

Key statistical tests used (Mock v RSV) using GraphPad Prism

Figure	*Array 1*	*Array 2*	*N*	*Test used*	*P value*	*95% Confidence interval*
**2A**	Mock 1h	RSV 1h	6	One tailed paired T Test	**0.0356**	-2782 to 47120
**2A**	Mock 4h	RSV 4h	6	One tailed paired T Test	0.0507	-
**2B**	Mock 1h	RSV 1h	6	One tailed paired T Test	**0.0034**	-3332 to 69622
**2B**	Mock 4h	RSV 4h	6	One tailed paired T Test	**0.0009**	51869 to 129873
**2C**	Mock 1h	RSV 1h	15	One tailed paired T Test	**<0.0001**	-1374 to -704.2
**2C**	Mock 4h	RSV 4h	15	One tailed paired T Test	**0.0011**	194.6 to 712.8
**2D**	Mock 1h	RSV 1h	15	One tailed paired T Test	**<0.0001**	-2328 to -1198
**2D**	Mock 4h	RSV 4h	15	One tailed paired T Test	**0.0052**	164.7 to 1037
**3B**	Mock 1h	RSV 1h	15	One tailed paired T Test	0.1418	-
**3B**	Mock 4h	RSV 4h	15	One tailed paired T Test	**<0.0001**	-13.19 to -5.252
**3C**	Mock 1h	RSV 1h	15	One tailed paired T Test	0.0507	-126.2 to 1256
**3C**	Mock 4h	RSV 4h	15	One tailed paired T Test	**<0.0001**	1102 to 2741
**3D**	Mock 1h	RSV 1h	4	One tailed paired T Test	0.4575	-
**3D**	Mock 4h	RSV 4h	4	One tailed paired T Test	**0.0177**	-0.3163 to -0.02174
**4A**	Mock 1h	RSV 1h	4	One tailed paired T Test	**0.0052**	-0.324 to -0.09367
**4A**	Mock 4h	RSV 4h	4	One tailed paired T Test	**0.0241**	-2.187 to -0.01673
**4B**	Mock 1h	RSV 1h	4	One tailed paired T Test	0.1654	-
**4B**	Mock 4h	RSV 4h	4	One tailed paired T Test	**0.0394**	-2.986 to 0.2875
**4C**	Mock 1h	RSV 1h	4	One tailed paired T Test	**0.0006**	-0.00315 to -0.00185
**4C**	Mock 4h	RSV 4h	4	One tailed paired T Test	0.4035	-
**4D**	Mock 1h	RSV 1h	4	One tailed paired T Test	0.0776	-
**4D**	Mock 4h	RSV 4h	4	One tailed paired T Test	0.0610	-
**4E**	RSV NM	RSV M	3	One tailed paired T Test	0.071	**-**
**4E**	ASM NM	ASM M	3	One tailed paired T Test	0.078	**-**
**4F**	RSV	RSV+N	9	One tailed paired T Test	**0.0263**	-55458 to 400.7
**4G**	RSV	RSV+N	10	One tailed paired T Test	0.4124	**-**

Key statistical tests used (RSV + RSV LFA1) using GraphPad Prism

Figure	*Array 1*	*Array 2*	*N*	*Test used*	*P value*	*95% Confidence interval*
**5A**	Mock 1h	RSV 1h	15	Two way ANOVA with Bonferroni correction	**<0.0001**	-2458 to -1068
**5A**	RSV 1h	RSV+LFA 1h	15		**<0.0001**	1125 to 2515
**5A**	Mock 4h	RSV 4h	15	Two way ANOVA with Bonferroni correction	0.1081	-
**5A**	RSV 4h	RSV+LFA 4h	15		**0.0091**	-1581 to -191.4
**5B**	RSV 1h	RSV+LFA 1h	4	Two way ANOVA with Bonferroni correction	0.878	-
**5B**	RSV 4h	RSV+LFA 4h	4		>0.99	-
**5D**	Mock 4h	RSV 4h	15	Two way ANOVA with Bonferroni correction	**0.0048**	521.7 to 3322
**5D**	RSV 4h	RSV+LFA 4h	15		**0.0323**	-2902 to -101.9
**5E**	Mock 4h	RSV 4h	15	Two way ANOVA with Bonferroni correction	**0.0002**	-14.34 to -4.232
**5E**	RSV 4h	RSV+LFA 4h	15		**0.0014**	2.832 to 12.94
**5G**	RSN+N	RSV+N+LFA	9	Wilcoxon matched-pairs signed rank test	0.9102	-

Statistical modelling using STATA

To validate our statistical tests were appropriate we carried out further analysis of the neutrophil adherence data using STATA. The results of this analysis are shown below:

Linear mixed models, allowing for clustering by donor, ratio scale

Histogram Adherence at 1 hour - ratio scale (not normally distributed)

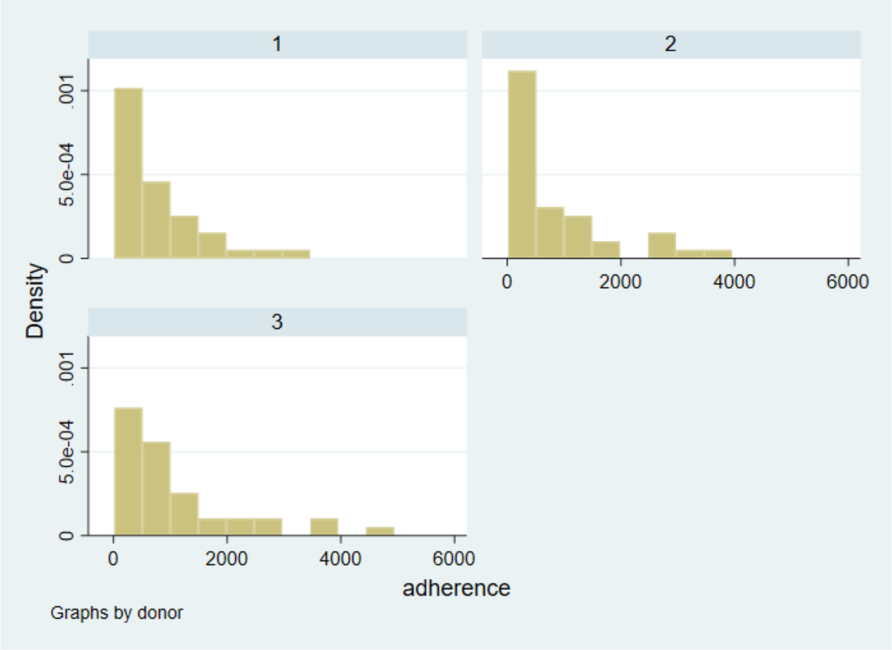

Linear mixed models, allowing for clustering by donor, log scale

Histogram Adherence at 1 hour - log scale (normally distributed)

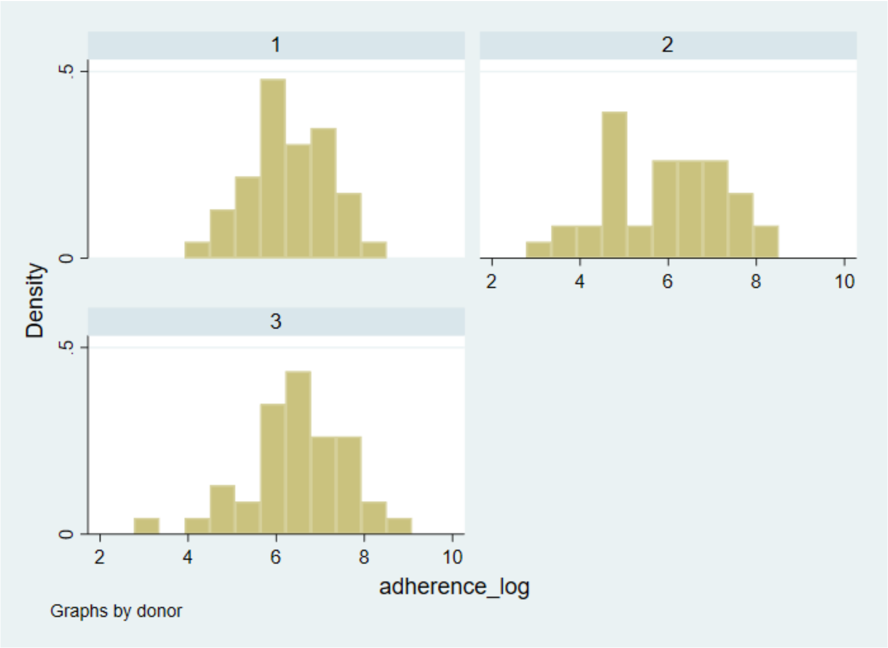

T test of normally distributed data

adherence_~g	Coef.	Std. Err.	z	P>z		[95% Conf.	Interval]
type_n							
M_R	0.190656	0.278341	0.68	0.493		-0.35488	0.736193
RSV	1.114467	0.278341	4.00	0.000		0.56893	1.660005
fMLP	-1.07402	0.278341	-3.86	0.000		-1.61955	-0.52848
_cons	6.669411	0.19814	33.66	0.000		6.281063	7.057759
sigma_u	0.039606						
sigma_e	0.762267						
rho	0.002692	(fraction	of variance due		to	u_i)	
