# Supplementary material for: β2-integrin LFA1 mediates airway damage following neutrophil transepithelial migration during respiratory syncytial virus infection
Source: Eur Respir J. 2020 Aug 6;56(2):1902216. doi: 10.1183/13993003.02216-2019 (PMC7406857; doi:10.1183/13993003.02216-2019)
Supplement: Supplementary file 7 [file ERJ-02216-2019.Shareable.pdf]

ORIGINAL ARTICLE
BASIC SCIENCE

SHAREABLE PDF

β_2-integrin LFA1 mediates airway damage following neutrophil transepithelial migration during respiratory syncytial virus infection

Jenny Amanda Herbert[1], Yu Deng[1,2], Pia Hardelid[1], Elisabeth Robinson[1], Luo Ren[1,2], Dale Moulding[1], Rosalind Louise Smyth[1,3] and Claire Mary Smith[1,3]

Affiliations: [1]UCL Great Ormond Street Institute of Child Health, London, UK. [2]Dept of Respiratory Medical Centre, Chongqing Key Laboratory of Child Infection and Immunity, Children's Hospital of Chongqing Medical University, China International Science and Technology Cooperation Base of Child Development and Critical Disorders, Ministry of Education Key Laboratory of Child Development and Disorders, Chongqing, China. [3]Joint senior author.

Correspondence: Claire Mary Smith, UCL Great Ormond Street Institute of Child Health, 30 Guilford St, London WC1N 1EH, UK. E-mail: c.m.smith@ucl.ac.uk

@ERSpublications
Neutrophils reduce RSV load, but their adherence to airway epithelial cells *via* β_2-integrin LFA1 inflicts collateral airway damage http://bit.ly/38ZOIn7

Cite this article as: Herbert JA, Deng Y, Hardelid P, *et al*. β_2-integrin LFA1 mediates airway damage following neutrophil transepithelial migration during respiratory syncytial virus infection. *Eur Respir J* 2020; 56: 1902216 [https://doi.org/10.1183/13993003.02216-2019].

This single-page version can be shared freely online.

ABSTRACT Respiratory syncytial virus (RSV) bronchiolitis is the most common cause of infant hospital admissions, but there is limited understanding of the mechanisms of disease, and no specific antiviral treatment. Using a novel *in vitro* primary transepithelial neutrophil migration model and innovative imaging methods, we show that RSV infection of nasal airway epithelium increased neutrophil transepithelial migration and adhesion to infected epithelial cells, which is associated with epithelial cell damage and reduced ciliary beat frequency, but also with a reduction in infectious viral load.

Following migration, RSV infection results in greater neutrophil activation, degranulation and release of neutrophil elastase into the airway surface media compared to neutrophils that migrated across mock-infected nasal epithelial cells. Blocking of the interaction between the ligand on neutrophils (the β_2-integrin LFA-1) for intracellular adhesion molecule (ICAM)-1 on epithelial cells reduced neutrophil adherence to RSV-infected cells and epithelial cell damage to pre-infection levels, but did not reduce the numbers of neutrophils that migrated or prevent the reduction in infectious viral load.

These findings have provided important insights into the contribution of neutrophils to airway damage and viral clearance, which are relevant to the pathophysiology of RSV bronchiolitis. This model is a convenient, quantitative preclinical model that will further elucidate mechanisms that drive disease severity and has utility in antiviral drug discovery.

Copyright ©ERS 2020. This version is distributed under the terms of the Creative Commons Attribution Licence 4.0.
